# An Update on the Molecular Mechanism of the Vertebrate Isthmic Organizer Development in the Context of the Neuromeric Model

**DOI:** 10.3389/fnana.2022.826976

**Published:** 2022-03-24

**Authors:** Matías Hidalgo-Sánchez, Abraham Andreu-Cervera, Sergio Villa-Carballar, Diego Echevarria

**Affiliations:** ^1^Departamento de Biología Celular, Facultad de Ciencias, Universidad de Extremadura, Badajoz, Spain; ^2^Instituto de Neurociencias de Alicante, Universidad Miguel Hernández-CSIC, Alicante, Spain

**Keywords:** midbrain, hindbrain, Otx2, Gbx2, engrailed, PAX, Fgf8, Wnt1

## Abstract

A crucial event during the development of the central nervous system (CNS) is the early subdivision of the neural tube along its anterior-to-posterior axis to form neuromeres, morphogenetic units separated by transversal constrictions and programed for particular genetic cascades. The narrower portions observed in the developing neural tube are responsible for relevant cellular and molecular processes, such as clonal restrictions, expression of specific regulatory genes, and differential fate specification, as well as inductive activities. In this developmental context, the gradual formation of the midbrain-hindbrain (MH) constriction has been an excellent model to study the specification of two major subdivisions of the CNS containing the mesencephalic and isthmo-cerebellar primordia. This MH boundary is coincident with the common *Otx2*-(midbrain)/*Gbx2*-(hindbrain) expressing border. The early interactions between these two pre-specified areas confer positional identities and induce the generation of specific diffusible morphogenes at this interface, in particular FGF8 and WNT1. These signaling pathways are responsible for the gradual histogenetic specifications and cellular identity acquisitions with in the MH domain. This review is focused on the cellular and molecular mechanisms involved in the specification of the midbrain/hindbrain territory and the formation of the isthmic organizer. Emphasis will be placed on the chick/quail chimeric experiments leading to the acquisition of the first fate mapping and experimental data to, in this way, better understand pioneering morphological studies and innovative gain/loss-of-function analysis.

## Early Development of the Central Nervous System: The Neuromeric Theory

**Figure 1:**
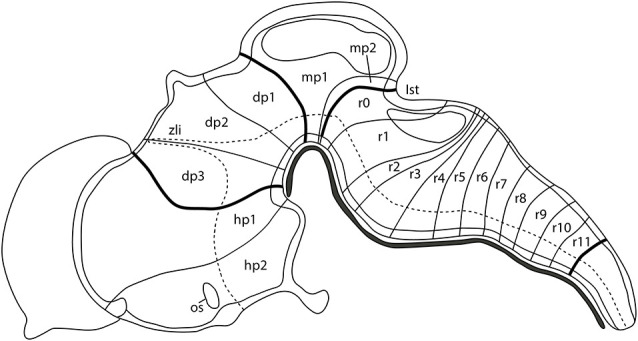
Schematic representation showing the neuromeric units of the developing mouse brain along its anterior-to-posterior axis: two hypothalamo-telencephalic prosomeres (hp1, hp2), three diencephalic prosomeres (dp1, dp2, dp3), two midbrain prosomeres (mp1, mp2), and 13 hindbrain rhombomeres (r0-r11). The dotted line defines the limit between the alar and basal plate of the developing neural tube. Ist, isthmus; os, optic stalk; zli, zona limitans intrathalamica. Adapted from Puelles (2018).

The complex central nervous system (CNS) of a vertebrate is a result of both ontogenic and evolutionary events. The first morphological evidence of the organization of the developing neural tube was reported by Orr (1887) introducing the term *neuromeres* as morphogenetic units arranged along its anterior-to-posterior axis and separated by transversal constrictions. In the chick embryos, classical anatomical studies showed that the incipient neuromeric units grouped in three primordial vesicles at the 10–12 somites stage (Vaage, 1969), representing the anlagen of the forebrain (secondary prosencephalon and diencephalon), midbrain (M; mesencephalon, mes), and hindbrain (H; classically divided in metencephalon, met, and myelencephalon, although nowadays these terms are considered obsolete). These main divisions will be further subdivided in the aforementioned neuromeres to produce a common complexity in all analyzed vertebrates according to the neuromeric model proposed by Luis Puelles’ group (Figure 1; Puelles, 2018). It is worth mentioning that the secondary prosencephalon contains the hypothalamo-telencephalic prosomeres (hp1 and hp2), whereas the diencephalon presents three diencephalic prosomeres (dp1, dp2, and dp3). In the same manner, the midbrain is divided into two mesomeres (mp1 and mp2) and the hindbrain is alienated into 13 rhombomeres clustered in: prepontine cryptorhombomeres (istmus, r0, and rostral and caudal parts of r1, r1r and r1c), pontine overt rhombomeres (r2-r4), retropontine overt rhombomeres (r5-r6), and medullary cryptorhombomeres (r7-r11; Figure 1; Puelles, 2018).

To obtain this conserved arrangement along the longitudinal axis of the brain, the development of the vertebrate neural tube is conducted by multi-step mechanisms, involving inductive and morphogenetic events. Long- and short-range diffusible molecules, morphogens, govern cell survival and fate specification through the control of dynamic spatial-temporal expression patterns of key transcription factors, which provides positional identities. Innovative genoarchitectural studies and fate mapping studies established correlations between how this intricate genetic network modulates the specification of neuroepithelial territories in vertebrate CNS (Puelles and Rubenstein, 2003; Puelles and Ferran, 2012; Puelles, 2018). According to the expression patterns of significant regulatory genes, such as *Otx2*, *Shh*, *Nk2.2*, and *Pax7*, and the distribution of dopaminergic and serotonergic neurons, among other molecular and cellular characteristics, the above-mentioned neuromeres could be grouped into three broad areas or tagmatic regions: forebrain (sum of the secondary prosencephalon, diencephalon, and midbrain), hindbrain, and spinal cord (Puelles, 2018). Our intention in this review is to focus on summarizing and discussing evidence concerning classical morphological and genetic views in the formation of the interface between the rostralmost tagma (forebrain) and the intermediate one (hindbrain), i.e., the interface between the caudal midbrain and the rostral hindbrain, the so-named midbrain-hindbrain (MH) boundary [This border is also referred to in classical studies as mesencephalic-metencephalic (mes-met) constriction]. The MH boundary, defined by the juxtaposition of the *Otx2*-expressing (rostral) and the *Gbx2*-expressing (caudal) domains in which the double decreasing gradients of *Engrailed* and *Pax2* expressions are centered, is associated with complex regulatory activities of a secondary organizer center, the isthmic organizer (IsO), mediated mainly by FGF8 and WNT1, two relevant morphogenes involved in the specification of the MH domain, including the meso-isthmo-cerebellar territory.

## The Mesencephalic/Metencephalic Constriction

One of the main goals in traditional embryology was to establish a model system to fate map the anterior-to-posterior arranged regions of the developing neural tube according to the proposed neuromeric model. The chick/quail chimeric system, introduced by Le Douarin (1969, 1973) has been a very useful approach to identifying cell derivatives from a small grafted portion of tissue. Using immuno-histochemical staining, the grafted cells from a donor quail embryo were easily identified when they were integrated with a host chick embryo. Alvarado-Mallart and Sotelo (1984) carried out innovative work to study the mesencephalic/metencephalic development using this well-designed experimental approach.

**Figure 2:**
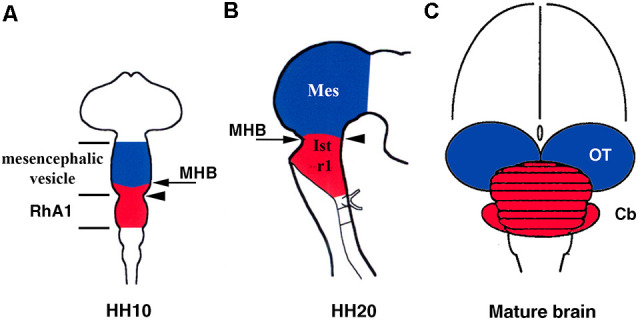
Location of the isthmocerebellar (red) and mesencephalic (blue) anlages in the chick neural tube at stage HH10 **(A)** and HH20 **(B)** with respect to the midbrain/hindbrain boundary (MHB; arrows in **A,B**). The arrowheads in **(A)** and **(B)** point to the mesencephalic/metencephalic (mes/met) constriction. Note that at stage HH20, the MH boundary and the mes/met constriction are coincident **(B)**, but not at stage HH10 **(A)**. The cerebellum (red) and mesencephalon (blue) are also shown in the mature brain **(C)**. Cb, cerebellum; Ist, isthmus (r0); Mes, mesencephalon; OT, optic tectum; RhA1, pro-rhombomere A1; r1, rhombomere 1. From Hidalgo-Sánchez et al. (2005a).

Furthermore, early anatomical observations in birds suggested that, at the 10-somite stage (HH10), the cerebellar primordium could be raised from the pro-rhombomere A1 (RhA1), the rostral most neuromere of the developing hind brain at this developmental stage, located therefore just caudal to the so-called mesencephalic/metencephalic (mes/met) constriction. This clearly defined vesicle of the developing neural tube would contain the presumptive territories of r1 and r2 (Vaage, 1969). Alvarado-Mallart’s group provided clear evidence of the location of the cerebellar primordium at this developmental stage (Figure 2A). Surprisingly, chick/quail homotopic grafts of the dorsal half of the mesencephalic vesicle at the stage HH10 showed that, in addition to the optic tectum, the isthmic nuclei and the rostromedial portion of the cerebellum raised from the grafted territory (Martinez and Alvarado-Mallart, 1989). Therefore, the “mesencephalic” vesicle observed at stage HH10 is the apparent midbrain vesicle that also includes the anterior portion of the hindbrain in chick embryos. The contribution of the “mesencephalic” vesicle to the cerebellum was also confirmed by Le Douarin’s group (Hallonet et al., 1990). At stage HH10, the isthmocerebellar primordium is, therefore, located more rostral than previously supposed, on both sides of the “mesencephalic/metencephalic” (“mes/met”) constriction (Figure 2A). Therefore, the final position of the cerebellum in the rostralmost portion of the hindbrain must be determined by important morphogenetic movements as development proceeds. At least in chick embryos, the cerebellum originates from several morphogenetic units (Figure 2; Martinez and Alvarado-Mallart, 1989; Hallonet et al., 1990; Alvarez-Otero et al., 1993; Marín and Puelles, 1995; Millet et al., 1996; reviewed in Alvarado-Mallart, 2000; Hidalgo-Sánchez et al., 2005a; Puelles, 2018).

## *Otx2* Confers Anterior Positional Identity in the Developing CNS

**Figure 3:**
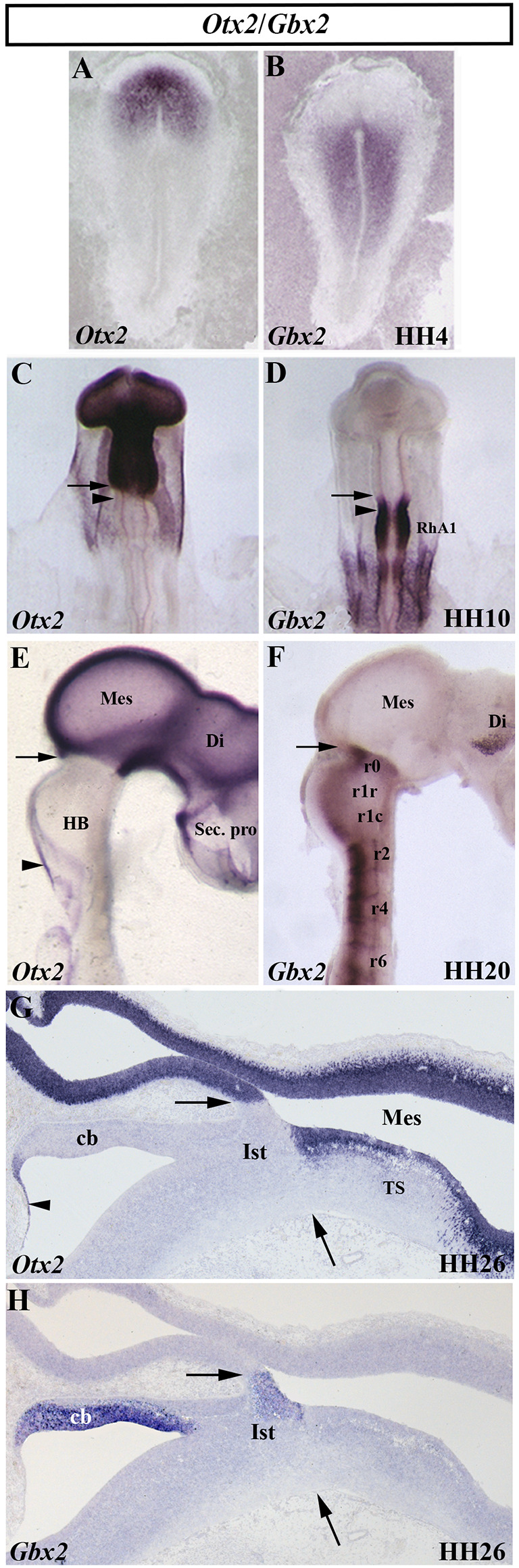
Spatial and temporal distribution of *Otx2* and *Gbx2* transcripts in the chick developing midbrain-hindbrain (MH) domain at different developmental stages. Single *in situ* hybridization performed in whole-mount **(A–F)** and in sagittal sections **(G,H)**. Note that, at all analyzed stages, the *Otx2*- and *Gbx2*-expressing domains are contiguous and complementary: stage HH4 **(A,B)**, HH10 **(C,D)**, HH20 **(E,F)**, and HH26 **(G,H)**. The arrows point to the MH boundary **(C–H)**, whereas the arrowheads in **(C)** and **(D)** point to the mes/met constriction. *Otx2* expression is observed in the mesencephalon (Mes), diencephalon (Di), and secondary presencephalon (Sec. pro; **E** and **G**). *Otx2* staining is also detected in the choroidal tissue (arrowheads in **E,G**). Different levels of *Gbx2* expression are detected in the rhombomeres (r) of the developing hindbrain (HB) at HH10 and HH20 stages **(D,F)**. Note the presence of *Gbx2* expression in the isthmus (Ist) and cerebellum (Cb) at stage HH26 **(H)**. RhA1, pro-rhombomere A1; TS, torus semicircularis. From Hidalgo-Sánchez et al. (1999a, 2005a,b).

Over the last decades, numerous efforts have been made to identify genes controlling programs of fate determination in the progressive subdivision of the neural tube along its anterior-to-posterior axis. The *Drosophila orthodenticle* (*otd*) gene and its vertebrate homolog (*Otx*) are clearly involved in the correct early specification of the anterior part of the developing head (Simeone et al., 1993; Finkelstein and Boncinelli, 1994). In mice, the expression of the *Otx2* gene, a homeobox-containing gene, is detected in the epiblast and the embryonic visceral endoderm before the onset of gastrulation and in the anterior neuroectoderm at the end of gastrulation stages (Acampora et al., 2001; Simeone et al., 2002; Kurokawa et al., 2004). A similar *Otx2* expression pattern was described in other vertebrate embryos (chick: Bally-Cuif et al., 1995; Figure 3A; *Xenopus*: Pannese et al., 1995; zebrafish: Mori et al., 1994; Mercier et al., 1995; Kesavan et al., 2017). *Otx2*^−/−^ mutant mice die early in development and show defects in anterior neuroectoderm specification. As a consequence, all analyzed mutant mice displayed an absence of the rostralmost tagmatic domain: secondary prosencephalon, diencephalon, and midbrain. In some phenotypes, an atypical hindbrain morphology, resembling the spinal cord, or even a deletion rostral to r3 were also observed, probably due to the mixed genetic background of progenitors (Acampora et al., 1995; Matsuo et al., 1995; Ang et al., 1996; Suda et al., 1997; Rhinn et al., 1998). In addition, studies of *Otx1*/*Otx2* mutant mice strongly suggest that *Otx* share functional similarities, being the dosage of *Otx* gene responsible for the correct anterior patterning of the developing brain (Acampora et al., 1997, 1998, 1999; Simeone et al., 2002; Kurokawa et al., 2004). In zebrafish, *Otx* loss-of-function embryos using morpholinos against two of the three zebrafish *Otx* genes (Mercier et al., 1995) gastrulate normally displaying a reduction of midbrain and an anterior shift of the isthmic tissue, together with an enlarged cerebellum, confirming that *Otx* is a repressor of cerebellar fate (Foucher et al., 2006). Therefore, *Otx* genes would govern the development of the secondary prosencephalon, diencephalon, and midbrain through the region-specific expression patterns of homeobox and cell adhesion genes (Rhinn et al., 1999).

**Figure 4:**
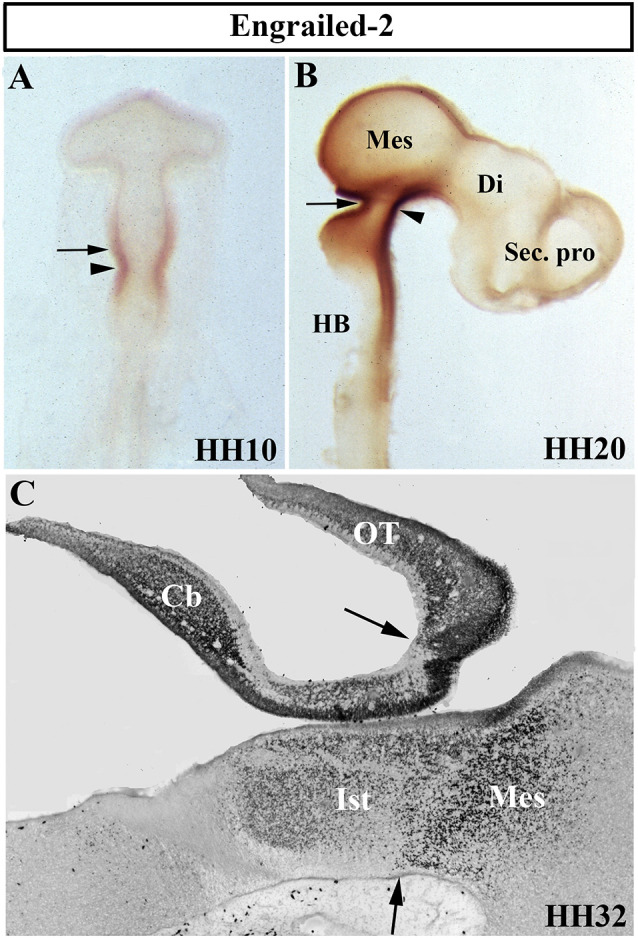
Distribution of Engrailed-2 (En2) at stages HH10 **(A)**, HH20 **(B)**, and HH32 **(C)** using immunoreaction with mAb 4D9 antibody in whole mount embryos **(A,B)** and in a sagittal section **(C)**. The arrowheads in **(A)** and **(B)** point to the mes/met constriction. En2 staining is observed on both sides of the MH boundary (arrows in **A–C**), in a territory which includes the prospective territories of the cerebellum (Cb), isthmus (Ist), and mesencephalon (Mes), the latter developing in part the optic tectum (OT). Di, diencephalon; HB, hindbrain; Sec. pro (secondary prosencephalon). From Hidalgo-Sánchez et al. (2005a).

In birds, analysis of whole-mount *in situ* hybridization with the *Otx2* probe (Figures 3C,E,G), anti-β-tubulin immunostaining as a marker of the first postmitotic neurons of the caudal midbrain, and chick/quail chimeras with various types of homotopic grafts analyzed after shorter and longer survival times showed that: (1) the caudal limit of *Otx2* expression is curved, forming a caudalwards “beak”, which coincides perfectly with the caudal border of the “mesencephalic” territory; (2) a transient *Otx2*-negative domain was observed in the caudalmost portion of the “mesencephalic” vesicle between stages HH10 and HH17/18, which gradually reduces its size until it disappears completely from stages HH17/18 (Figure 3C); (3) so that, the caudal limit of the *Otx2* expression coincides with the constriction observed at stage HH20, but not before (Figure 3E); (4) the *Otx2*-negative territory detected at stage HH10 in the caudal “mesencephalic” vesicle will give rise to isthmic nuclei and the mediorostral cerebellum (Figure 3G) as shown in long-survival chimeras; and (5) similar embryonic events were also observed in mouse embryos. Rostrocaudal morphogenetic movements take place in the meso-isthmo-cerebellar domain between stages HH10 and HH17/18. Therefore, the constriction observed in the meso-isthmo-cerebellar domain at stages 10 and 20 is not the same entity (Figures 2A,B; Millet et al., 1996; Alvarado-Mallart, 2000; Garda et al., 2001; Hidalgo-Sánchez et al., 2005a).

## *Gbx2* Is Involved in the Anterior Hindbrain Specification

The gastrulation brain homoebox (*Gbx*) genes, vertebrate homologs of the unplugged (*unp*) gene of *Drosophila*, are also directly involved in the specification of the MH domain (Wassarman et al., 1997; Broccoli et al., 1999; Hidalgo-Sánchez et al., 1999a, b; Li et al., 2002; Kikuta et al., 2003; Rhinn et al., 2003, 2004, 2009; Sunmonu et al., 2009, 2011; Burroughs-Garcia et al., 2011; Su et al., 2014; Tsuda et al., 2019). In the chick, a detailed analysis showed a dynamic expression of the *Gbx2* gene, together with the *Otx2* gene, at early developmental stages (Figure 3). At stage HH4, *Otx2* and *Gbx2* expressions are observed in the rostral and caudal portion of the chick embryo, respectively (Figures 3A,B). While the expressing domains of both genes are not contiguous at stage HH8, with a small gap of expression between them, a slight overlap is observed at stage HH9 (Garda et al., 2001). At stage HH10, the *Otx2*-expressing domain and the *Gbx2*-expressing domain are contiguous and exclusive (Figures 3C,D). As expected, the *Otx2*/*Gbx2* common limit is far from the “mest/met” constriction a stage HH10. However, it is coincident with the constriction observed at stage HH20 (Figure 2; Millet et al., 1996; Hidalgo-Sánchez et al., 1999a, 2005a; Garda et al., 2001). Although some differences could be observed (Bulfone et al., 1993; Su and Meng, 2002; Rhinn et al., 2003), the posterior border of *Otx2* expression and the anterior border of *Gbx2* expression are coincident and labeled the MH boundary in all analyzed vertebrates (Figures 3C–H; Bouillet et al., 1995; von Bubnoff et al., 1996; Niss and Leutz, 1998; Hidalgo-Sánchez et al., 1999a; Garda et al., 2001; Tour et al., 2001; Pose-Méndez et al., 2016). Therefore, the role of the *Gbx2* gene in the anterior hindbrain specification could be conserved across evolution (Castro et al., 2006). It is worth remarking that *Otx2-* and *Gbx2-* expressing domains are firstly established independently of each other, with antagonistic interactions between them as development proceeds to create the *Otx2*/*Gbx2* MH interface (Figure 3; Li and Joyner, 2001).

In mice, *Gbx2* is essential for the precise location of the MH boundary (Millet et al., 1999; Sunmonu et al., 2009, 2011). A histological study of *Gbx2* mutant embryos showed the absence of a cerebellum, which is formed in normal conditions by distinct vermal, hemispheric, and floccular portions that coincide with the three prepontine units from which it originates (r0, r1r, and r1c; Puelles, 2018). The isthmic nuclei and IV motor nucleus (derived from isthmus; r0), locus coeruleus (r1), and V motor nucleus (r2/3) were absent in mutant homozygotes. Derivatives from the hindbrain caudal to r3 were normal under histological analysis. Thus, the VII motor nucleus (r4/5) displayed normal development in the correct places. Interestingly, *Hoxb1* expression, a marker of r4, was observed close to the midbrain caudal end, whereas *Krox20*, a marker of r3 and r5, was exclusively present in its caudal domain (r5). Therefore, the *Gbx2* mutant shows a relevant alteration of derivatives of r1–3, giving rise to a shortened area with abnormal histological characteristics. In the caudal midbrain, the III motor nucleus (midbrain) developed in the correct position. However, the midbrain displays abnormalities in its anterior/posterior patterning, suggesting that the caudal midbrain, inferior colliculi, could suffer a caudal extension to the r3/r4 border according to a caudal shift of the *Otx2* domain (Wassarman et al., 1997).

The analysis of a conditional mouse mutant using the *Cre/loxP* system and lacking *Gbx2* expression in r1 after E8.5 showed that these *Gbx2*-CKO embryos displayed no apparent alterations in motor coordination and developed cerebellum with variable defects only in the vermis, this medial region showing a disrupted foliation pattern (Li et al., 2002). Interestingly, a similar defect of the cerebellar vermis was found in mice that express *Otx2* in r1 from the *En1* locus (*En1*^+/*Otx2*^; Broccoli et al., 1999). The cerebellar hemispheres of *Gbx2*-CKO embryos displayed a normal development, the cytoarchitecture of the entire cerebellum being completely normal. Also, the inferior colliculi seemed slightly enlarged, suggesting a posterior shift of the MH boundary location. Of interest, strong ectopic *Otx2* expression was found at E14,5 in abnormal cell aggregates near the ventricular layer of the *Gbx2*-CKO cerebellum, which likely is involved in altering vermis development (Li et al., 2002).

In addition, *Gbx2* seems to have a permissive role in r3 specification (Li and Joyner, 2001), ectopic *Gbx2* expression in *Hoxb1*-*Gbx2* transgenic mice not being enough to induce r1–3 development in r4 (Li et al., 2005). It seems to be possible that a *Gbx* dosage (*Gbx1* and *Gbx2*) requirement could be necessary for the correct development of specific rhombomeres in the rostral hindbrain (Waters and Lewandoski, 2006). In this sense, some *Otx2* deficient mice also failed to form a normal brain rostral to r3 (Acampora et al., 1995; Matsuo et al., 1995; Ang et al., 1996). Besides, *Gbx2* activity could confine *Otx2* expression by binding to its FM enhancer (Inoue et al., 2012). These findings strongly suggest mutual *Otx2*/*Gbx2* interactions for correct MH domain development by means of the establishment of the precise *Otx2*/*Gbx2* common border and its consequence inductive events (see below).

In zebrafish, *gbx* genes play a redundant role in morphogenesis and differentiation of the cerebellum (Kikuta et al., 2003; Rhinn et al., 2003, 2004, 2009; Su et al., 2014). *gbx2* is very relevant in MH specification in zebrafish embryogenesis in a dose-dependent manner. Injections of a low dose of *gbx2* mRNA cause exclusively a less evident MH constriction, with a normal anterior brain, while injections of high doses provoked repression of regional markers of the secondary prosencephalon, diencephalon, and midbrain and a strong disruption of these brain areas (Nakayama et al., 2013). Also, ectopic *gbx2* expression by mRNA injection in other works confirmed the loss of these three areas of the zebrafish brain and caused a severely altered rostral hindbrain, isthmus, and cerebellum (Kikuta et al., 2003), with an increased cell death in r2/3 and r5 and disorganized nerve V neurons (r2/3; Burroughs-Garcia et al., 2011). Similar results were obtained in *Xenopus* with overexpression of *Xgbx2a* or *Xgbx2b* genes (Tour et al., 2002a). A shortening of the anterior hindbrain was also detected in zebrafish *gbx1*-;* gbx2*- double mutants, produced by a progressive loss of the genetic program leading to MH specification (Su et al., 2014), with a phenotype similar to or less severe than that observed in mutant mice (Wassarman et al., 1997). In summary, the establishment of the *Otx2*/*Gbx* common boundary is mediated by mutual inhibitory interactions between these two genes at early developmental stages in all analyzed vertebrates. Concerning the molecular evolution of this regulatory mechanism, the duplication-degeneration complementation (DDC) model, based on complementary degenerative mutations in a pair of duplicated genes, was used to elucidate how the mechanism diverged between tetrapod and teleost for vertebrate *Gbx* genes (Islam et al., 2006).

## The Meso-Isthmo-Cerebellar Region Expresses High Levels of *En* Genes

The *Drosophila engrailed* gene is essential for accurate embryonic segmentation and neurogenesis in the developing insects, its conserved sequence and biochemical function being identified in a high number of organisms throughout the animal kingdom. The members of the homeobox-containing gene *En* family (*En1* and *En2*), homologs to the *Drosophila engrailed* gene (Joyner et al., 1985; Joyner and Martin, 1987), is the first gene whose expression pattern was described in the developing MH domain of all analyzed vertebrate embryos, both genes showing a similar expression pattern (Davidson et al., 1988; Davis and Joyner, 1988; Gardner et al., 1988; Patel et al., 1989; Davis et al., 1991; Hemmati-Brivanlou et al., 1991; Ekker et al., 1992; Logan et al., 1992). Focused on chick embryos, *En2* expression starts to be observed at stage HH9 (Figure 4A), its high expression levels being coincident with the prospective cerebellar anlagen (Martinez and Alvarado-Mallart, 1989). At stage HH19–20, the En2-staining domain forms a double decreasing gradient (Figure 4B) in a broad area around the *Otx2*/*Gbx2* MH boundary, this En2-positive area including the presumptive territory of the cerebellar, isthmic, and mesencephalic anlagen (Figure 4C; Gardner et al., 1988; Davis et al., 1991; Logan et al., 1992; Millet and Alvarado-Mallart, 1995).

*En1* null mutant mice died soon after birth with a great number of developmental alterations, in particular a relevant loss of both cerebellar and mesencephalic structures. The mesencephalic III (oculomotor) and rhombencephalic IV (trochlear) cranial nerves were lacking in homozygous mutant embryos. This phenotype may be caused by regional control of cell precursor proliferation instead of by cell fate determination; there is a loss or lack of proliferation of mid-hindbrain cells in *En1* mutants (Wurst et al., 1994). However, ectopic cell death was also detected during the development of the prospective midbrain and cerebellum in *En1* null homozygotes (Chi et al., 2003). *En2* mutant mice are viable and display a relatively mild phenotype with some motor control problems as a consequence of the slight reduction in cerebellar size, a disturbed cerebellar foliation pattern in the caudal cerebellum, and a delay in the fusion of the cerebellar rudiments at the midline, together with smaller colliculi (Joyner et al., 1989, 1991; Millen et al., 1994). Therefore, the analysis of both *En1* and *En2* homozygous mutant mice suggests a partially redundant function of these genes in MH domain specification (Hanks et al., 1995). However, the comparison of the phenotypes of a varied combination (conditional, knock-in, and null) of *En1/2* double-mutant mice clearly showed a differential requirement for En1 and En2 proteins in the specification of subdomains in the developing cerebellum and tectum (Sgaier et al., 2007). In a very significant way, the analysis of double *En1/En2* CKO mutants and single CKOs showed that both *En1* and *En2* genes act together and are essential to guarantee the foliation patterning of the developing vermis and hemispheres by regulating the spatial restriction of key gene expressions, as well as to ensure the correct position and formation over time of fissures in the vermis, these genes acting late in embryogenesis (Cheng et al., 2010). In any case, *En2* has a greater potential to control foliation than *En1* (Sgaier et al., 2007). In summary, an “engrailed code” could be necessary for a correct dose-dependent subdivision of the tectal and cerebellar primordiain antero-to-posterior arranged functional subdomains (Sgaier et al., 2007; see also Simon et al., 2005 and Sgado et al., 2006 for other derivatives from MH domain).

In the tectal chick anlagen, *En2* expression shows a decreasing gradient from caudal (stronger) to rostral (weaker). The works of Nakamura’s group have shown that En2 determines the exact position of the rostral mesencephalic boundary by repressing diencephalic markers, such as Pax6, and activating mesencephalic genes (Araki and Nakamura, 1999). Also, En2 confers posterior positional information in the developing tectum for tectal polarity formation and retinotopic projections. Using the chick/quail transplantation experimental model at the 10-somite stage, heterotopic grafts of the mesencephalic alar plate into the diencephalon showed a reverse *En2* expression pattern, i.e., a rostrocaudal decreasing gradient from the mes-diencephalon border (Itasaki et al., 1991), and the topographic order of retinal fiber projections in these chimeric embryos changes in concordance with the inverted gradient of *En2* expression (Itasaki and Nakamura, 1992; see Itasaki and Nakamura, 1996 for retroviral gene experiments). In addition, the prosencephalon differentiates into an optic tectum when transplanted into the mesencephalon, showing a typical laminar pattern and optic nerve fibers from the eyes extending to it (Nakamura et al., 1991). Similar results were obtained when the optic tectum was rotated in quail-chick chimeric embryos (Matsuno et al., 1991). Furthermore, *En2* is involved in the tectal laminar formation, the rostral part of the optic tectum showing more advanced lamination than the caudal one, as a potential regulator of neuronal cell migration (Omi et al., 2014). In summary, these findings strongly suggest that *En2* expression is controlled by environmental influences, governing the rostro-caudal polarity of the optic tectum (Friedman and O’Leary, 1996; Logan et al., 1996; Shigetani et al., 1997; Bilovocky et al., 2003; reviewed by Omi and Nakamura, 2015).

## *Pax2/5/8* Genes in the Midbrain-Hindbrain Specification

**Figure 5:**
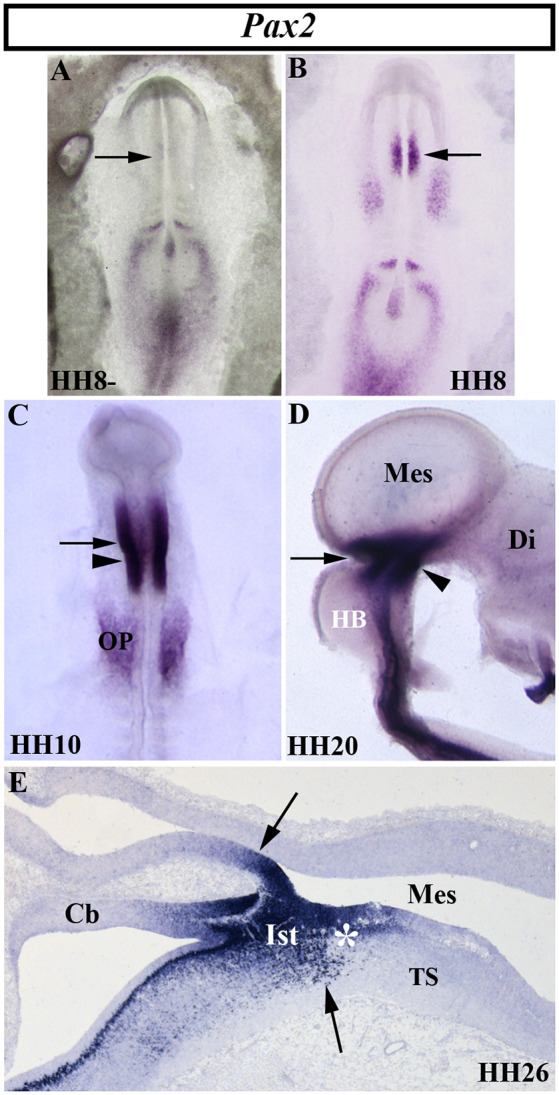
Spatial and temporal distribution of *Pax2* transcripts in the chick developing MH domain at different developmental stages. Single *in situ* hybridization performed in whole-mount **(A–D)** and in sagittal sections **(E)**. In the presumptive MH domain, *Pax2* expression starts to be detected at stage HH8, but not before (arrows in **A,B**). At stages HH10, HH20, and HH26, *Pax2* transcripts are detected on both sides of the MH boundary (arrows in **C,E**). The arrowheads in **(C)** and **(D)** point to the mes/met constriction. At stage HH26 **(E)**, *Pax2* expression is observed in the isthmic (Its) and pre-isthmic (asterisk in **E**) domains. Cb, cerebellum; Di, diencephalon; HB, hindbrain; Mes, mesencephalon; OP, otic placode; TS, torus semicircularis. From Hidalgo-Sánchez et al. (1999a, 2005b).

The vertebrate paired-box-containing *Pax* gene family, ortholog of the *Drosophila Prd* gene, encodes transcription factors which were highly conserved during evolution. This gene family is involved directly in animal body plan, regulating morphogenesis, organogenesis, and cell differentiation. These genes are also clearly implicated in the neuromeric organization of the brain because of their restricted expression patterns along the anterior-to-posterior axis of the developing neural tube. In particular, the *Pax2/5/8* subfamily is expressed in the MH domain during ontogenesis with possible functional redundancy and equivalency between them (Krauss et al., 1991; Asano and Gruss, 1992; Stoykova and Gruss, 1994; Urbánek et al., 1994; Rowitch and McMahon, 1995; Heller and Brändli, 1997; Schwarz et al., 1997; Pfeffer et al., 1998; Rowitch et al., 1999; Goode and Elgar, 2009; Namm et al., 2014; Kesavan et al., 2017 among others). In chick embryos (Figure 5), *Pax2* transcripts are detected in a portion of the developing neural tube on both sides of the MH junction at stages HH10 and HH20 (Figures 5C,D; Okafuji et al., 1999; Hidalgo-Sánchez et al., 1999a, 2005b). *Pax2* expression is previous to *Pax5* expression, labeling a smaller area included in the broader *Pax5*-positive domain (Funahashi et al., 1999). Thus, both *Pax2*- and *Pax5*-expressing domains form double rostral and caudal decreasing gradients, centered on the common *Otx2*/*Gbx2*MH boundary, similar to *En2* expression (Figures 3–5). The area of overlap of *Otx2* and *Pax2* expressions in the caudal midbrain defines the preisthmic domain, contiguous to the MH boundary (Figures 3G; 5E). In both birds and mammalians, this histogenetic domain contains a caudal part of the midbrain reticular formation, the interfascicular nucleus, and the magnocellular (pre) isthmic nucleus, as well as the matching part of the periaqueductal gray (Hidalgo-Sánchez et al., 2005b; Puelles et al., 2013). These studies settle the four distinct midbrain histogenetic domains present in the midbrain alar plate: griseum tectale (rostrally), the optic tectum, the torus semicircularis, and the preisthmic domain (caudally; Hidalgo-Sánchez et al., 2005b).

In mice, *Pax2* expression is observed at E7.5, also previous to *Pax5* expression, the latter occurring at E8.25 (Asano and Gruss, 1992; Urbánek et al., 1994; Rowitch and McMahon, 1995). The homozygous *Krd* mutation, a deletion in chromosome 19 resulting in the whole deletion of *Pax2* locus, is lethal at the preimplantation stage. Deletion of one *Pax2* allele in *Krd^(+/–)^* mutant mice exhibited a normal development of the MH domain (Urbánek et al., 1997). On the other hand, *Pax5* homozygous mutants display a weak brain phenotype with slight morphological alterations in the posterior midbrain (inferior colliculus) and a perturbation of the cerebellum foliation pattern (Urbánek et al., 1994). Interestingly, *Pax5 ^(+/–)^ Krd^(+/–)^* mouse embryos show a significant absence of the caudal midbrain and alteration of the cerebellar vermis, whereas *Pax5^(−/−)^ Krd^(+/–)^* embryos display a complete loss of the midbrain and the rostral hindbrain (Urbánek et al., 1997). Similar morphological alterations were reported for the spontaneous *Pax2*^1Neu^ mouse mutation (Favor et al., 1996). Other works showed that one allele of *Pax2*, but not *Pax5*, is necessary and sufficient for MH development in the absence of *Pax5*, these genes not being subjected to cross-regulation by each other (Schwarz et al., 1997). The possible discrepancies could be caused by the different genetic backgrounds used. In the zebrafish, alterations of *Paz[b]* gene function confirm the evidence from mouse embryos. Injection of neutralizing antibodies raised against the Paz[b] protein (Krauss et al., 1992), the *noi* (*noi*stmus) mutation (Brand et al., 1996; Lun and Brand, 1998; Pfeffer et al., 1998), or the homozygous Tg [pax2a-hs:eGFP] embryos (Ota et al., 2016) also result in the loss of the MH domain. All these findings strongly suggest the regulatory interaction between *Pax2* and *Pax5* genes in the accurate development of the MH domain by the activation of common target genes according to a critical dose-dependent effect of Pax proteins (Brand et al., 1996; Schwarz et al., 1997, 1999; Urbánek et al., 1997; Bouchard et al., 2005). Interestingly, *Pax5 ^(−/−)^ Krd ^(+/–)^* mutant mice display an absence of *En1/2* expressions (Urbánek et al., 1997) and their phenotypes are similar to that reported in *En1* mutant mice (Wurst et al., 1994). Thus, *Pax* genes could cooperate with other early markers of the MH domain to assure MH fate (Tallafuß and Bally-Cuif, 2003).

Using *in ovo* electroporation for gain-of-function experiments, it was reported that *Pax2* plays a relevant role in the first step of midbrain specification, whereas *Pax5* could sustain the state of midbrain differentiation once it is committed (Funahashi et al., 1999; Okafuji et al., 1999). *Pax2* ectopic expression in the diencephalon transformed it into a mesencephalon after *En2* induction and *Pax6* repression, the latter being a diencephalic marker (Okafuji et al., 1999; Matsunaga et al., 2000). Although, *Pax2* and *Pax5* genes could be higher hierarchical elements in the genetic cascades devoted to the specification and determination of the midbrain primordium (Lun and Brand, 1998; Funahashi et al., 1999; Bouchard et al., 2005; Goode and Elgar, 2009), a mutual regulation between En and Pax2 functions has also been suggested (Picker et al., 2002). Different enhancers with functional *Pax2/5/8*-biding sites in mouse *Pax* genes could govern the activation and maintenance of their expressions in the MH domain at different developmental stages (Pfeffer et al., 2000, 2002). The existence of redundant regulatory elements in members of the *Pax* gene family could explain their mutual cooperation in MHB specification (Song et al., 1996).

## An Organizer Center Is Present in the Central Portion of the Mid-Hindbrain Domain

**Figure 6:**
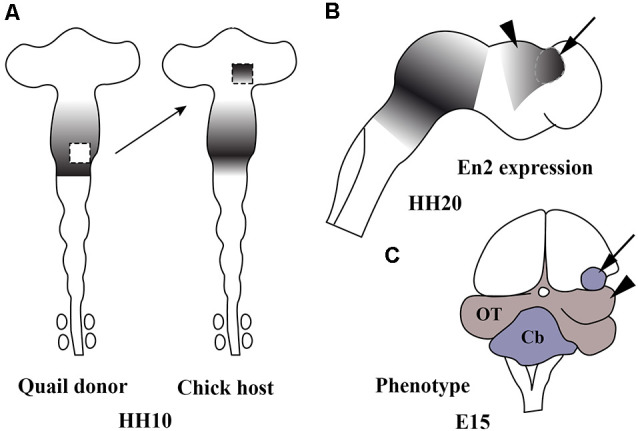
Schematic representation of chick/quail chimeric experiments showing the ectopic induction of En2 in the alar plate of the caudal diencephalon of chick (host; **A**) when the isthmocerebellar primordium of quail (donor; **A**), expressing high levels of En2, was grafted into it at stage HH10 (Martinez et al., 1991). The En2-expressing area is represented by gray areas. At stage HH20 **(B)**, the quail graft (arrow) maintains its original En2 expression and induces an inverted gradient of En2 expression within the caudal prosencephalon (arrowhead), typical of the mesencephalic vesicle. A long-survival analysis performed at El5 **(C)** shows that the graft (arrow) developed a small cerebellum and the En2-induced host primordium changed its fate for an ectopic mesencephalon (arrowhead). Cb, cerebellum; OT, optic tectum. From Alvarado-Mallart (2000).

The central portion of the mid-hindbrain domain expressing high levels of *En1/2* and *Pax2/5* could be considered the first organizer center described in the developing neural tube. Using the chick/quail chimeric experimental model, the alar plate of the quail isthmocerebellar primordium was transplanted ectopically to the caudal chick diencephalon (dp1 and dp2) at the 10-somite stage (Figures 6A,B; HH10), i.e., restricted in an area just caudal to the zona limitans intratalamica (Martinez and Alvarado-Mallart, 1990; Martinez et al., 1991). The graft maintained the original *En2* expression and kept its cerebellar and isthmic nuclei fate. Interestingly, the quail-grafted *En2*-positive territory was able to induce a caudal decreasing gradient of *En2* expression in the contiguous chick diencephalon (Figure 6C), which never expresses *En2* under normal conditions (Figure 4B). The host *En2*-positive induced tissue developed an ectopic supernumerary midbrain (Figure 6C), showing the laminar cytoarchitectonic structure of an optic tectum (Martinez et al., 1991). Both supernumerary generated structures in the host, the grafted isthmocerebellum and the induced mesencephalon, displayed an inverted rostral-to-caudal order and orientation with respect to the meso-isthmo-cerebellar structures observed in the normal MH domain (Martinez and Alvarado-Mallart, 1990; Martinez et al., 1991; reviewed by Alvarado-Mallart, 1993, 1997, 2000). A conserved regulatory mechanism was also observed between mammals and birds (Martinez et al., 1991). These results agree with those from *En2* ectopic-induced experiments in the caudal diencephalon concomitant with a *Pax6* repression (Bloch-Gallego et al., 1996; see also Okafuji et al., 1999; Matsunaga et al., 2000).

In addition, the caudal diencephalon (dp1 and dp2), but not the dp3 and the secondary prosencephalon, was competent to express *En2* and changed its phenotype to be adapted to the integration side when it was transplanted in close contact with the *Otx2*/*Gbx2* MH boundary, from where the mentioned double *Pax2*/*En2* decreasing gradients originated (Martinez et al., 1991; Bloch-Gallego et al., 1996). Thus, the caudal-grafted diencephalon is able to develop isthmic and cerebellar structures, as well as an optic tectum. These phenotypic changes never take place when the rostral mesencephalic vesicle is considered in the experimental model, which displays low levels of *En2* expression (Gardner and Barald, 1991). It is worth noting that the rostral mesencephalic vesicle transplanted into the prosencephalon at the 10-somite stage lost its original *En2* expression, while the caudal mesesenphalic vesicle maintained *En2* expression (Gardner and Barald, 1991). Therefore, different regulatory/inductive properties could be considered between the rostral and caudal halves of the mesencephalic vesicle observed at stage HH10 (Alvarado-Mallart et al., 1990).

**Figure 7:**
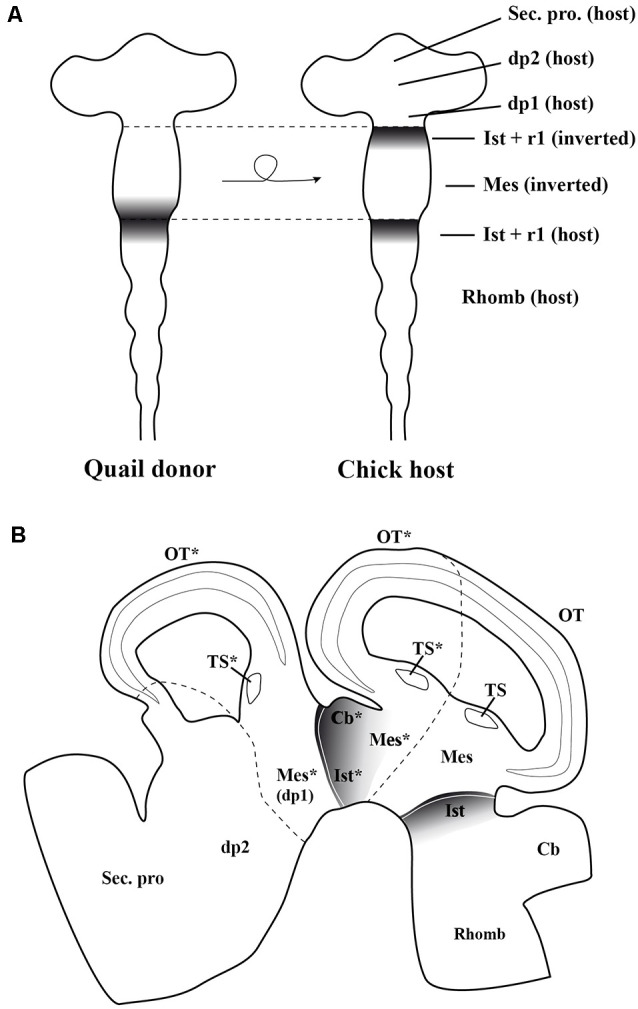
**(A)** Schematic representation of the chick/quail chimeric experiments in which the quail (donor) “mesencephalic” vesicle at stage HH10, containing both the mesencephalic primordium and the rostral part of the isthmocerebellum, was grafted into a chick (host) embryo after inversion of its rostrocaudal axis (Marín and Puelles, 1995). The En2-expressing area is represented by gray areas. In the resulting chimeric embryo, the grafted isthmocerebellum was positioned just in contact with the host pd1 (diencephalon), whereas the rostra1 “mesencephalic” vesicle was in direct interaction with the staying host pro-rhombomere 1, RhA1. **(B)** Sagittal section of the resulting chimeric embryo in a long-survival analysis. The grafted-rotated portion of the neural tube is located between both dotted lines. The rostral and caudal portions of isthmocerebellum kept its original phenotype giving rise to the cerebellum (Cb) and isthmic nuclei (Ist). The grafted mesencephalic primordium formed a bicaudal phenotype, as showed by the symmetry of its cytoarchitecture organization of the optic tectum (OT) and a duplication of the torus semicirculais (TS). The host dp1 changed its fate for a complex caudal mesencephalic structure (OT plus TS), the phenotype of the contiguous dp2 not being perturbed. Asterisks, ectopic structures observed in experimental studies. Mes, mesencephalon; r1, rhombomere 1; Rhomb, rhombecephalon; Sec. pros, secondary prosencephalon. Adapted from Marín and Puelles (1995).

These inductive properties were studied in detail analyzing all MH neuronal structures generated after the inversion of a portion of the neural tube containing the presumptive midbrain plus its caudally adjacent territory, the isthmocerebellar primordium (Figure 7A; Marín and Puelles, 1994). The analysis of short-survival chick/quail chimeric embryos showed that the *En2* expression pattern of the inverted portion was abnormal, displaying two mirror-duplicated areas of high *En2* expression on both its rostral and caudal halves. As expected, the caudal diencephalon was induced to express the *En2* gene. Cytoarchitecture analysis of long-survival resulting chimeras showed that the inverted graft developed an inverted isthmocerebellar structure in its rostral aspect plus an altered midbrain whose rostral and caudal halves show a caudal alar mesencepahilic grisea phenotype with symmetrical morphology and polarity, the rostral alar mesenphalic area (the griseum tectale) being absent (Figure 7B). The contiguous caudal diencephalon changed its fate drastically to an isthmic and caudal mesencephalic phenotype with a normal polarity with respect to the entire brain axis (for more anatomical details concerning the alar and basal plates, see Marín and Puelles, 1994). Experiments in which the inversion of the portion of the neural tube containing exclusively the prospective mesencephalon, without any portion of the isthmocerebellar prospective domain, were also performed. In these chimeric cases, the grafted neuroepithelium showed a normal pattern of *En2* expression in short survival analysis, while the torus semicircularis, optic tectum, and griseum tectalis formation presented a normal caudal-to-rostral order in long survival studies. Thus, these findings fit well with the existence of a source of polarizing signal placed in the prospective isthmocerebellar domain, located just in the caudal most portion of the “mesencephalic” vesicle observed at stage HH10 (Martinez and Alvarado-Mallart, 1989; Crespo-Enriquez et al., 2012).

The possible plasticity of the entire rhombencephalon with respect to the *En2*-expressing MH boundary was also studied. When transplanted into the rhombencephalon, the grafted “mesencephalic” vesicle differentiates into an optic tectum, without any structure resembling a cerebellar cytoarchitecture (Nakamura, 1990). However, the presumptive territory of the isthmocerebellum maintained always its *En2* expression and cerebellar phenotype, and also induced a fate change in the hindbrain; it induced ectopically *En2* expression in the competent rhombomeres, but caused the development of a cerebellum with a typical cytoarchitectural profile in the host contiguous neural tissue instead of an optic tectum (Martínez et al., 1995). It is interesting to remark that, in this kind of experiment, the observed inductive consequences did not cross the boundaries between adjacent rhombomeres (clonal restriction; Martínez et al., 1995). However, the prospective isthmic area, grafted in the posterior hindbrain, caudal to the rhombomere 8, or in the spinal cord was not able to induce *En2* expression and to develop a cerebellar-like structure in host tissue, the grafted area changing its phenotype to adapt to its new environment (Grapin-Botton et al., 1999).

The competence of developing a neural tube to express the *En2* gene has been studied. In the chick, a great portion of the developing neural tube at stage HH10, extending from the dp2 to r7, is able to express *En2* and to change its prospective fate. The induced phenotype appears to depend on its position in relation to the “mes/met” constriction. When a neuromere is situated rostral to the “mes/met” constriction, the *En2*-induced tissue develops a mesencephalic phenotype, whereas caudal to it, it develops a cerebellar structure (Alvarado-Mallart, 2000; Hidalgo-Sánchez et al., 2005a).

## FGF8 Is the Molecular Effector of the Isthmic Organizer Activity

**Figure 8:**
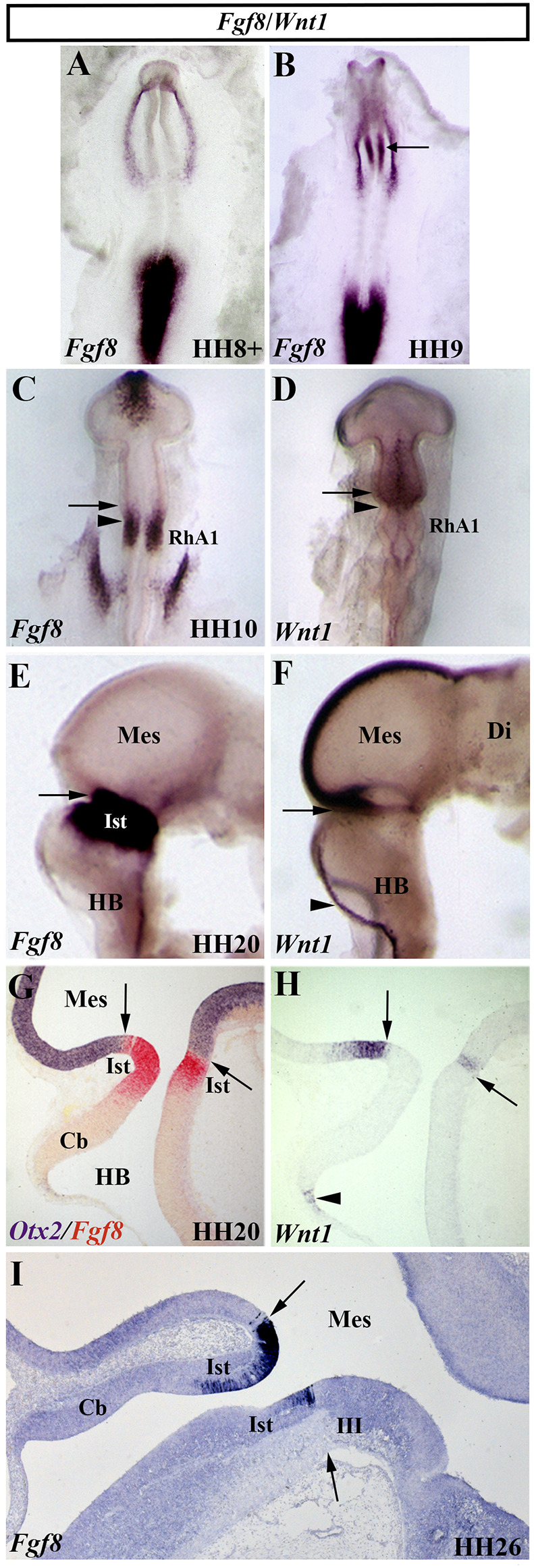
Spatial and temporal expression of *Fgf8* and *Wnt1* genes in the chick developing MH domain at different developmental stages. Single **(A–F,H,I)** and double **(G)** *in situ* hybridization performed in whole-mount **(A–F)** and in sagittal sections **(G–I)**. The arrows point to the *Otx2*/*Gbx2* MH boundary. In the developing MH domain, *Fgf8* transcripts are first detected at stage HH9 (**A**; arrow in **B**). At stage HH10, *Fgf8* expression is observed in a portion of the neural tube just caudal to the *Otx2*/*Gbx2* MH boundary (arrow in **C**) and rostral to the mes/met constriction (arrowhead in **C**). *Wnt1* expression is detected in the entire “mesencephalic” vesicle, rostral to the mes/met constriction (arrowhead in **D**), with a stronger expression in its dorsal midline **(D)**. At stage HH20, *Fgf8* and *Wnt1* expressions are present on both sides of the *Otx2*/*Gbx2* MH boundary (arrows in **E–H**), as confirmed by double *Otx2* and *Fgf8* staining (arrow in **G**). *Wnt1* transcripts are also detected in the choroidal tissue (arrowheads in **F**,**H**). Note that the isthmic region (Ist, r0) is clearly *Fgf8* positive at stages HH20 **(E,G)** and HH26 **(I)**. Cb, cerebellum; Di, diencephalon; HB, hindbrain; III, trochlear nucleus; Mes, mesencephalon; RhA1, pro-rhombomere 1. From Hidalgo-Sánchez et al. (1999a); Hidalgo-Sánchez et al. (2005a, 2005b).

The gene family of the fibroblastic growth factors (*Fgf*) is required in embryonic development leading to growth and patterning events (Delgado and Torres, 2017; Thawani and Groves, 2020). The *Fgf8* gene, firstly identified by encoding a secreted, androgen-induced signaling molecule (Tanaka et al., 1992), is one of the most relevant signaling molecules with long-range diffusible activity. The well-defined spatial and temporal expression pattern of the *Fgf8* gene in the prospective isthmic domain clearly proposes the FGF8 as the main inductive molecule emanating from it and controlling all the previously mentioned developmental events taking place in the MH domain of all analyzed vertebrates (mouse: Heikinheimo et al., 1994; Ohuchi et al., 1994, 2000; Crossley and Martin, 1995; Bueno et al., 1996; chick: Figures 8A–C,E,G,I; Hidalgo-Sánchez et al., 1999a; Ohuchi et al., 2000; Garda et al., 2001; *Xenopus*: Christen and Slack, 1997; zebrafish: Reifers et al., 1998; reviewed by Echevarria et al., 2005; Vieira et al., 2010). In the chick, *Fgf8* expression is first detected at stage HH9 (Figures 8A,B), much later than *Otx2* and *Gbx2* expressions (Figures 3A,B). At stage HH10, the *Fgf8*-expressing domain is observed in a portion of the neural tube containing the caudal most portion of the “mesencephalic” vesicle and the rostral half of the RhA1 (Figure 8C), that is on both sides of the so-named “mes/met” constriction (Figure 2A; Hidalgo-Sánchez et al., 1999a; Garda et al., 2001). Remarkably, this *Fgf8*-expressing area is located just in a narrow transversal band where *Otx2*-positive and *Gbx2*-positive domains overlap (Garda et al., 2001). At stage HH14–15, when the *Otx2* and *Gbx2* co-expression disappears and both *Otx2*-expressing and *Gbx2*-expressing domains are mutually exclusive and complementary, i.e., when the *Otx2*/*Gbx2* midbrain/hindbrain boundary is rightly established, the *Fgf8*-positive area overlaps the rostralmost portion of the *Gbx2*-positive domain, where the prospective isthmus develops, abutting the *Otx2*-positive domain (see Figures 8E,G,I for stages HH20 and HH26, respectively; Hidalgo-Sánchez et al., 1999a, 2005b; Garda et al., 2001).

The isthmus, also named r0, is the rostralmost segmental unit of the hindbrain (Aroca and Puelles, 2005; Puelles, 2013). Using *Fgf8*-Cre-LacZ mice to determine the cell derivatives from the *Fgf8* expressing domain and anatomical characteristics, the Puelles’s group defined the isthmus as a distinct transverse segment of the mammalian brain delimited rostrally by the *Otx2*/*Gbx2* boundary and the MH constriction, whereas caudally the r0/r1 boundary is defined exclusively by the borders of regulatory gene expression patterns such as *Irx2* (Watson et al., 2017). Therefore, the r0/r1 could be considered a cryptic (molecular) boundary. In mice, the isthmus contains the same derivatives which have been previously described in birds (Puelles et al., 2007). In addition to contributing to the formation of the cerebellar vermis (the rostralmost portion of the cerebellum), the developing isthmic domain in birds contains the avian parvicellular and semilunar isthmic nuclei (homologous to the parabigeminal and microcellular tegmental nuclei in mammals), the trochlear nucleus (whose cranial motor root emerges from the dorsal isthmic surface), the dorsal nucleus of the lateral lemniscus, the caudal linear nucleus, a rostral part of the interpeduncular nucleus, and the caudal isthmic part of the substantia nigra, among other alar and basal derivatives (for more anatomical details, see Alvarez-Otero et al., 1993; Aroca et al., 2006; Puelles et al., 2007; Lorente-Cánovas et al., 2012; Alonso et al., 2013; Watson et al., 2017). Although some isthmic neurons migrate into the contiguous neuromeres, the caudal midbrain and r1, these findings do not question the existence of lineage restriction in r0 (Watson et al., 2017).

FGF8 is the molecule responsible for the isthmic organizer properties of the isthmus or r0. Beads soaked with FGF8 recombinant protein, inserted in the caudal diencephalon or in the rostral mesencephalon, are able to induce ectopic *En1/2* expression and to change the fate of the insertion tissue into two ectopic, mirror-image mesencephalon and isthmic nuclei, as well as cerebellar structures with cells expressing CaBP, a Purkinje cell marker (Crossley et al., 1996; Martinez et al., 1999; Garda et al., 2001), mimicking the inductive effects obtaining through grafting experiments (Martinez and Alvarado-Mallart, 1990; Martinez et al., 1991). FGF8 induced *Fgf8* expression and repressed *Otx2* expression in the neuroepithelium around the implanted beads. Besides, FGF8-soaked beads also induced *Gbx2* expression in mouse explant (Liu et al., 1999). On the other hand, *Fgf8*-expressing isthmic grafts integrated with the caudal hindbrain or the spinal cord show a downregulation of its *Fgf8* expression together with a progressive loss of its inductive ability, the fate being adapted to the host environment (Grapin-Botton et al., 1999). In summary, FGF8-soaked beads and the resulting effects of ectopic-grafted isthmic organizer tissue strongly suggest that FGF8 is responsible for inductive events mediated by the prospective isthmic domain. In this genetic context, it is relevant to highlight that *Pax2* is crucial for the induction of *Fgf8* expression at the MH boundary (Ye et al., 2001). Loss-of-function studies in mice confirm the involvement of an FGF8 signaling pathway during gastrulation and in the induction and specification of the MH domain (Meyers et al., 1998; Sun et al., 1999; Chi et al., 2003). *Fgf8*^−/−^ mutant mouse development fails easily at gastrulation, these mice show a prospective neuroectoderm with crucial alterations (Sun et al., 1999).

According to the described *Fgf8*^−/−^ phenotypes in mice, the spatiotemporal action of a dose-dependent FGF8 activity could also regulate tissue specification of the tectal-isthmo-cerebellar structures in several periods of the embryonic periods (Basson et al., 2008; Sato and Joyner, 2009). A sustained and strong FGF8 action may control the formation of the structures next to the isthmus (part of the medial inferior colliculi, the isthmus, and the medial-anterior cerebellum), whereas low levels and brief FGF8 activity could be sufficient for the correct formation of structures far from it (the superior colliculi and the lateral cerebellum). It could involve a *Fgf8*-mediated *Otx2* repression and the maintenance of an accurate relationship among expression patterns of key regulatory genes in the proximity of the MH boundary. Other *Fgf* genes could also be involved. The reduced *Fgf17* expression observed in *Fgf8* temporal CKOs could explain the observed survival of most midbrain/r1 cells near the isthmus (Sato and Joyner, 2009).

In zebrafish, the *acerebellar* (*ace*) mutant embryos show an absence of isthmus and cerebellum, whereas an enlarged tectum is formed (Brand et al., 1996; Reifers et al., 1998; Picker et al., 1999; Jászai et al., 2003; Tallafuß and Bally-Cuif, 2003; see also Araki and Brand, 2001 for morpholino-induced knockdown of Fgf8), together with a loss of *eng* and *pax-b* expressions (Brand et al., 1996; Reifers et al., 1998). The observed tectal expansion was caused by caudal-to-rostral transformation, with a noticeable cell fate change and without perturbation of cell proliferation and apoptosis patterns. FGF8-soaked beads rescue MH development with normal isthmocerebellar structures, confirming the key role of FGF8 in patterning and polarizing the MH domain (Jászai et al., 2003). Also, these mutant embryos show a partial disruption of the retinotectal map compared to the wild type, these results suggesting that FGF8 from the isthmic area could mediate the establishment of midbrain polarity (Picker et al., 1999). However, early *Fgf8* expression does not require *pax2a* (Reifers et al., 1998), supporting the hypothesis of an early MH domain specification in at least two phases (Reifers et al., 1998; Tallafuß and Bally-Cuif, 2003). In the initial establishment phase in late gastrulation, midbrain and rostral hindbrain specification could be established in an independent way within an MH-pro-domain, probably depending on *Otx2* and *Gbx2* instructive regulatory activities. In a second maintenance phase, MH development could be governed by a complex regulatory network of signaling molecules generated all around the MH boundary, *fgf8* being mainly involved in the reinforcement of an r1 vs. r2 identity. Besides, zebrafish embryos with reduced *Otx* activity and without *fgf8* function (*fgf8*^−/−^; *OtxH* mutants) display an enlarged r1 but with some cerebellar cells, suggesting that neither the *fgf8* gene nor other members of the *Fgf* family could be required for r1 specification and cerebellar development, as well as for cerebellar cell differentiation. However, *fgf8* seems to be relevant to maintaining r1 devoid of *Otx2* expression defining the MH boundary, in addition to being necessary for cell proliferation and cerebellar primordium morphogenesis (Foucher et al., 2006). Interestingly, the locus coeruleus, originated in the alar plate of r1 (Aroca et al., 2006), was absent in these *fgf8*^−/−^; *OtxH* mutant embryos (Foucher et al., 2006).

The FGF signaling pathway is mediated *via* four tyrosine kinase-type transmembrane receptor (FGFR) proteins, which trigger several intracellular signaling cascades, including Ras-ERK pathways as a subclass of mitogen-activated protein kinase pathway (Echevarria et al., 2005). The expressions of the *Fgfrs* genes have been described in the developing nervous system of vertebrates, particularly in the midbrain and anterior hindbrain (Friesel and Brown, 1992; Yamaguchi et al., 1992; Walshe and Mason, 2000; Liu et al., 2003; Trokovic et al., 2003, 2005; Scholpp and Brand, 2004; Jukkola et al., 2006; Blak et al., 2007b; Saarimäki-Vire et al., 2007; Ota et al., 2010; Rohs et al., 2013; Leerberg et al., 2019). *Fgfr1* expression was present in the dorsal aspect of the MH domain (Friesel and Brown, 1992; Walshe and Mason, 2000; Trokovic et al., 2003, 2005; Scholpp and Brand, 2004). FGFR1 seems to be the main FGF receptor receiving FGF signals from the isthmic organizer (Trokovic et al., 2003, 2005; Jukkola et al., 2006), *Fgfr2* and *Fgfr3* being dispensable for normal MH development (Blak et al., 2007a). Thus, tissue-specific inactivation of *Fgfr1* in *En1-Cre*/+; *Fgfr1^flox/flox^* mice caused a failure of the MH constriction and a deletion of the posterior hindbrain (the posterior colliculi) and the anterior portion of the cerebellum (the vermis), the cerebellar hemispheres being present only with an altered foliation. Large deletions were also detected in the midbrain, mainly affecting the inferior colliculi. Similar effects were observed in mice homozygous for hypomorphic *Fgfr1* alleles. In contrast, the basal plate of the MH domain (the oculomotor and trochlear nuclei, as well as the locus coeruleus and the substance nigra) did not display any deletions or alterations in *En1-Cre*/+; *Fgfr1^flox/flox^* mice (Trokovic et al., 2003, 2005). Once the isthmic organizing center has been established, a sustained *Fgfr1* expression could be necessary for maintaining the response to the isthmic signals and for correct specific cell-adhesive characteristics to take place at the MH border (Trokovic et al., 2003).

Using a conditional gene inactivation method without any perturbed *Fgf8* expression during gastrulation, it has been shown that part of the FGF8 regulatory activity is due to its involvement in cell survival, associated with *En* and *Gbx2* activities, among other genes, the prospective midbrain being affected earlier than the cerebellum (Chi et al., 2003). Thus, cell death in the presumptive MH domain increases when the level of *Fgf8* expression decreases (Chi et al., 2003). This failure of cell survival was also confirmed at least in part in works with loss-of-function analysis of the *Fgf* signaling pathway (Basson et al., 2008; Sato and Joyner, 2009). In this sense, the *Lmx1b.1* and *Lmx1b.2* genes could participate in this cellular event by the regulation of *Fgf8* and *Wnt1* (O’Hara et al., 2005).

Surprisingly, *Fgfr1* inactivation does not affect cellular survival (Trokovic et al., 2003). However, the study of mice embryos with different combinations of *Fgfr1*, *Fgfr2*, and *Fgfr3* mutations showed a redundant participation of FGFRs in supporting cell survival, governing the anterior-to-posterior specification of the MH domain (Saarimäki-Vire et al., 2007).

## *Wnt1* Activity During MH Domain Development

The vertebrate *Wnt1* gene, homolog of the segment polarity *wingless* (*wg*) gene in *Drosophila* (Rijsewijk et al., 1987), encodes a short-range diffusible molecule involved in complex cell-cell signaling processes, governing several embryonic events (tissue patterning, cell fate determination, apoptosis, and proliferation, among others). In the developing neural tube of vertebrates, *Wnt1* expression is detected in the MH domain (mouse: Wilkinson et al., 1987; Bally-Cuif et al., 1992, 1995; Parr et al., 1993; chick: Bally-Cuif and Wassef, 1994; Hollyday et al., 1995; Sugiyama et al., 1998; Hidalgo-Sánchez et al., 1999a; zebrafish: Molven et al., 1991; Kelly and Moon, 1995; Lekven et al., 2003; Buckles et al., 2004; Xenopus: Wolda et al., 1993). In chick embryos, the *Wnt1* gene displays a dynamic expression pattern. At stage HH7, *Wnt1* transcripts are present in the presumptive mesencephalic area of the neural plate prior to the neural tube closure. At stage HH10, *Wnt1* expression is observed in the entire “mesencephalic” vesicle with a higher expression in its caudal half (Figure 8D). At this developmental stage, the caudal border of *Wnt1* expression extends slightly more caudal than the *Otx2*-positive domain, thus overlapping somewhat with the *Gbx2*-expressing domain (Figures 3C,D, Figure 8D; Hidalgo-Sánchez et al., 1999a). In stage-HH20 chick embryos, *Wnt1* expression is confined to a ring encircling the neural tube in the most caudal portion of the mesencephalic vesicle and on its dorsal midline, the latter extending rostral into the caudal prosencephalon until the prospective epiphysis (Figure 8F). *Wnt1* expression is also observed in the rhombic lip of the developing hindbrain (Figures 8F,H; Bally-Cuif and Wassef, 1994; Hollyday et al., 1995). At this developmental stage, double *in situ* hybridization showed that the encircling ring of *Wnt1* expression is located in the caudal most portion of the *Otx2*-positive mesencephalic domain, abutting dorsally with the *Fgf8*- and *Gbx2*-expressing domain (Figures 8G,H; Hidalgo-Sánchez et al., 1999a).

Using the chimeric experimental model, Bally-Cuif and co-workers showed that, when the caudal mesencephalic vesicle was transplanted into the prosencephalon, a chick *En2* induction was observed in the surrounding tissue together with *Wnt1* expression in the graft (Bally-Cuif et al., 1992; Bally-Cuif and Wassef, 1994). Of course, the grafted tissue included an *Fgf8*-expressing neuropithelium (Hidalgo-Sánchez et al., 1999a), therefore it is not possible to assign this inductive effect exclusively to WNT1 diffusible molecules. In this sense, *in vitro* assays showed that a selective antagonist of WNT signals or an inhibitor of FGFR activation can block the expression of MH markers in MH explants at stage 4, suggesting that WNT and FGF signaling pathways are required and sufficient for the initial induction of the isthmic activity at the gastrula stage in chick embryos (Olander et al., 2006). However, similar neural explant assays with tissues isolated from HH12 embryos showed that WNT signaling post-gastrulation is involved in maintaining the early pattern of *Fgf8* expression in the MH domain and, consequently, the isthmic identity (Canning et al., 2007). Therefore, it is not possible to confirm if WNT1 and FGF8 instructive actions are already present at the gastrula stage (Olander et al., 2006) or shortly thereafter (Canning et al., 2007).

Mouse embryos lacking *Wnt1* function fail to form an MH boundary with a severe reduction of the midbrain and no obvious cerebellum, the rostral midbrain juxtaposing directly with the caudal hindbrain (McMahon and Bradley, 1990). Although *En1* expression was apparently normal in 4-somite littermate homozygous for mutated *Wnt1* allele, the *En1*-expressing domain was completely lost in the MH domain at the 21–30-somite stage (McMahon et al., 1992). These anatomical abnormalities in the development of the midbrain and cerebellum were also confirmed by the Capechi’ group analyzing other less-severe *Wnt1* (*swaying/sw*) disruptions in mice (Thomas and Capecchi, 1990; Thomas et al., 1991). In *Wnt1^sw/sw^* embryos with a severe phenotype, the transverse *Wnt1^sw^*-expressing ring of the MH boundary was completely missing and ectopic patches of *Otx2*-expressing cells were detected in r1, without crossing the r1/2 border. These *Wnt1^sw/sw^* embryos showed an MH domain that was severely shortened. In *Wnt1^sw/sw^* embryos with mild phenotype, the *Wnt1^sw^* ring was interrupted and scattered patches of ectopic *Wnt1^sw^*-expressing cells were observed in the rostral r1, just in the rostralmost aspect of some ectopic *Otx2*-expressing patches of cells. In all analyzed *Wnt1^sw/sw^* embryos, both cranial nerve III (caudal most midbrain) and IV motoneurons (rostralmost hindbrain) were absent, which normally develop on both sides of the transverse *Wnt1*-positive ring. Therefore, the straight common border of *Otx2/Wnt1^sw^* expressions at the MH boundary failed to form correctly. In summary, *Wnt1* could be needed firstly for the specification of the MH domain, probably by the maintenance of *En* expression territory (McMahon et al., 1992; Wurst et al., 1994; Danielian and McMahon, 1996; Sugiyama et al., 1998) and later for the segregation of mesencephalic (*Otx2*-positive) and isthmocerebellar (*Otx2*-negative, and so *Gbx2*-positive) phenotypes. The latter effect could be mediated by correct adhesive properties of cell-cell interactions at the *Otx2*/*Wnt1* MH boundary (Bally-Cuif et al., 1995).

Some differences have been observed among chick, mouse, and zebrafish development (Lekven et al., 2003; Buckles et al., 2004). In zebrafish, the analysis of homozygous *wnt1/wnt10b* deficient embryos and morpholino antisense (MO)-mediated oligonucleotide knockdown showed that only a portion of the *pax2a* and *en2a*-expressing areas in the MH domain is dependent on the *wnt1* and *wnt10b* activities. In these experimental embryos, the resulting phenotype was almost normal with only faint defects (Lekven et al., 2003). Both *wnt1* and *wn10b* genes are not necessary for the standard maintenance of *fgf8* and *en2b* expressions, as well as those of other *wnt* genes (*wnt8b* and *wnt3a*). Therefore, the *wnt1* and *wn10b* genes seem not to be needed, surprisingly, for the correct development of the midbrain and cerebellum in zebrafish development (Lekven et al., 2003). Morpholinos knockdown in zebrafish also showed that *wnt3a* is required for the correct MH domain development in absence of *wnt1* and *wnt10b* activities, the expression of *en2*, *pax2a*, and *fgf8* not being maintained after mid-somitogenesis (Buckles et al., 2004). *wnt3*/*wnt1*/*wnt10b* deficient embryos suffer wide-ranging apoptosis in the prospective MH domain resulting in a major loss of the midbrain and cerebellum (Buckles et al., 2004). Because of the fact that *wnt* genes have self-governing and coinciding functions during zebrafish development, the possible function of other *wnt* genes, such as *wnt8b*, in the accurate development of the MH domain cannot be ruled out (Buckles et al., 2004; Rhinn et al., 2005).

### The Genetic Cascade at the Mid-Hindbrain Boundary

The juxtaposition of differently pre-specified areas could generate interfaces and specific diffusible morphogenes. The action of these long- or short-ranging molecules with possible antagonist effects could trigger specific genetic programs in a dose-dependent manner devoted to governing the specification of large domains by means of key regulatory genes (Meinhardt, 2009). In the developing MH domain, the specification of *Otx2*- and *Gbx2*-expressing confronting areas at earlier developmental stages could determine the precise location of the MH boundary. The co-regulation of two relevant signaling pathways, FGF and WNT, together with the action of transcription factors, such as PAX and EN, among others, could be responsible for the inductive events mediated by the isthmic organizer center (Broccoli et al., 1999; Hidalgo-Sánchez et al., 1999b; Irving and Mason, 1999; Martinez et al., 1999; Millet et al., 1999; Katahira et al., 2000; Garda et al., 2001; Li and Joyner, 2001; Martinez-Barbera et al., 2001; Matsunaga et al., 2002; Hidalgo-Sánchez and Alvarado-Mallart, 2002; Tour et al., 2002a, b; Panhuysen et al., 2004; Li et al., 2005).

**Figure 9:**
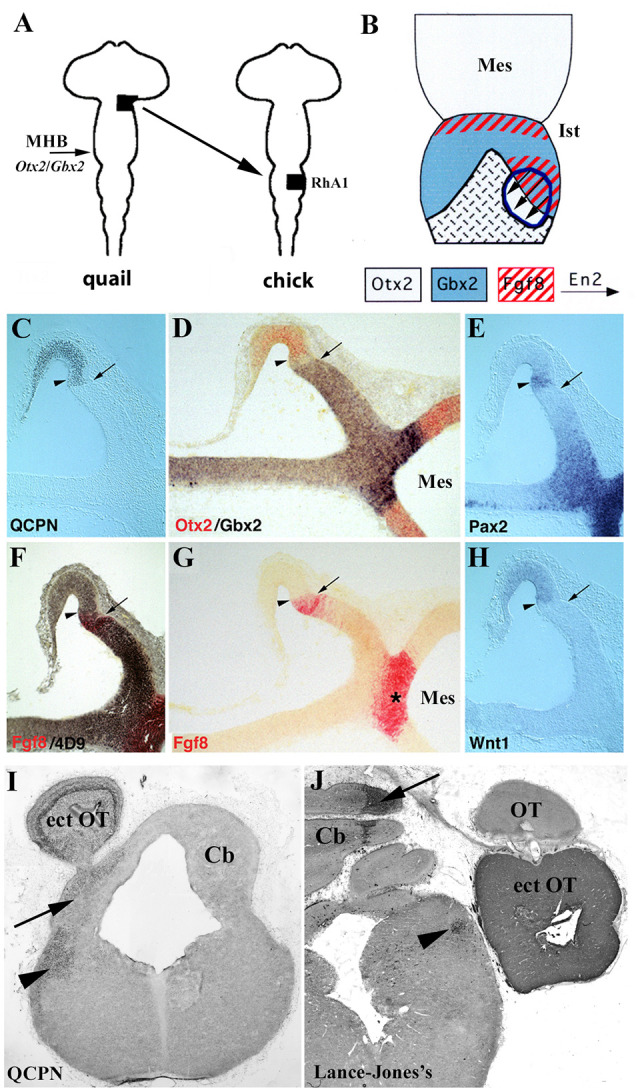
**(A)** Schematic representation of heterotopic transplant, black areas, of the quail (donor) dp1/dp2 neuroepithelium to the chick (host) pro-rhombomere A1 (RhA1), caudal to the mes/met constriction, performed at stage HH10. **(B)** Schematic representations of a dorsal view of short survival chimeric embryos. In light gray, *Otx2*-positive territories in the midbrain, in the choroid tissue, and in the graft. In blue, *Gbx2* positive domains. The red, *Fgf8*-positive territories. The arrows indicate the En2 induction. The graft is outlined by blue lines. A new *Otx2*/*Gbx2* boundary is formed within the graft. **(C–H)** Serial sagittal sections of a resulting chimeric embryo at stage HH20. The arrows point to the QCPN-positive graft/host interface and the arrowheads to the newly created intragraft *Otx2*/*Gbx2* boundary. *Pax2*, En2, *Fgf8*, and *Wnt*expressions display a pattern on both sides of the intragraft *Otx2*/*Gbx2* boundary similar to that observed in the host MH domain: the *Fgf8* and *Wnt1* genes are expressed within the *Gbx2* and *Otx2* domains, respectively, and *Pax2* and En2 (4D9 immunoreaction) form double decreasing gradients centered on this intragraft *Otx2*/*Gbx2* boundary. The *Fgf8*-induced area is undoubtedly separated from the host *Fgf8*-positive isthmic ring (asterisk in **G**). **(I,J)** Frontal sections of two long-survival chimeric embryos treated with QCPN **(I)** and Lance-Jones’s **(J)** antibodies, which labeled quail cells from the grafts. The transplants form an ectopic optic tectum (ect OT), also contributing to both* in situ* isthmus (Ist; arrowheads) and cerebellum (Cb; arrows). Mes, mesencephalon; MHB, midbrain/hindbrain boundary; OT, host optic tectum.

Pivotal studies using chick/quail chimeric systems carried out by the Alvarado-Mallart’s group are devoted to unveiling the role of the *Otx2*/*Gbx2* mutual interaction in the MH domain (Figure 9; Alvarado-Mallart, 2000; Hidalgo-Sánchez et al., 2005a). When the quail alar portion of the *Otx2*-expressing caudal diencephalon, competent to express *En2*, was transplanted at the 10-somite stage (HH10) into the chick MH territory, always in direct contact with *Gbx2-*expressing RhA1 (Figure 9A), the expression of *Gbx2* and *Fgf8* was induced in a small portion of the graft just contiguous to the host *Gbx2*-positive domain (Figure 9B). Interestingly, the original *Otx2* expression was repressed in the *Gbx2*/*Fgf8* induced domain within the transplant (Figures 9C,D). Indeed, a new *Otx2*/*Gbx2* interface was created within the grafted territory. The other genes involved in the specification of the MH domain (*Fgf8*, *Wnt1*, *En2*, and *Pax2*) showed expression patterns similar to those described in normal development: *Fgf8* and *Wnt1* were expressed in two narrow bands, while *Pax2* and *En2* formed double decreasing gradients centered on the new intra-graft *Otx2*/*Gbx2* boundary (Figures 9C–H; Hidalgo-Sánchez et al., 1999b). In the induced genetic event, *En2* and *Pax2* were the first genes to be expressed, while *Otx2* repression and *Gbx2* induction occurred later with an initial gap of *Otx2*/*Gbx2* expression as observed in normal development (Garda et al., 2001). *Fgf8* was the last gene to be expressed in the *Gbx2*-induced portion of the graft (Hidalgo-Sánchez and Alvarado-Mallart, 2002). A long-term survival analysis showed that the grafts developed a supernumerary optic tectum at the level of the host cerebellum from the *Otx2*/*En2*/*Pax2*/*Wnt1*-expressing area. This area also contributed to both *in situ* isthmus and cerebellum, which develops from the *Gbx2*/*En1–2*/*Pax2*/*Fgf8*-expressing one (Figures 9I,J; Hidalgo-Sánchez et al., 1999b, 2005a). It is also worth noting that the induction of *Fgf8* expression was also evident in experiments in which the prospective territories of the midbrain (*Otx2*-positive) and r1 (*Gbx2*-positive) were juxtaposed (Irving and Mason, 1999). The mutual repression of *Otx2* and *Gbx2* genes was also confirmed in gain-of-function experiments in chicks using *in ovo* electroporation. When *Otx2* was ectopically expressed in the rostral hindbrain, the alar plate of r1 differentiated into the optic tectum instead of an isthmocerebellar structure. In addition, when *Gbx2* was ectopically expressed in the mesencephalon, the *Otx2* caudal mesencephalic limit shifted rostrally. Thus, ectopic activations of *Otx2* and *Gbx2* induced *Fgf8* expression at the new *Otx2*/*Gbx2* interface (Katahira et al., 2000).

Knock-in *Otx2* in *En1* domain mice showed an *Otx2* overexpression in the *En1*-positive territory, which includes the prospective anterior hindbrain. In these experimental embryos, a posterior expansion of *Otx2* expression into rh1 was observed, whereas *Gbx2* expression was downregulated in the ectopic *Otx2*-expressing territory (Broccoli et al., 1999). Therefore, the*Otx2*/*Gbx2* border changed caudally towards a new position, together with the expression of associated genes (*Pax2*, *Fgf8*, and *Wnt1*). The histological analysis of these *En1^+/Otx2lacZ^* transgenic mice showed that the inferior colliculi were enlarged, prolonging caudally, whereas the cerebellum was smaller than in normal conditions with a loss of the anterior vermis and a deficiency of cerebellar midline fusion (Broccoli et al., 1999). In other kinds of experiments combining *Gbx2*-GOF and *En^Cre^* knock-in allele in mice, an ectopic *Gbx2* expression was also induced in the midbrain and in the rostralmost portion of the hindbrain (Sunmonu et al., 2009). Unexpectedly, the midbrain and cerebellum were truncated in the most severe phenotype of these *En^Cre^*^/+^; *Gbx2*-GOF mutant mice (Sunmonu et al., 2009), instead of a predictable enlarged cerebellum and a reduction of the midbrain (Millet et al., 1999; Katahira et al., 2000). *Fgf8* expression was significantly reduced to a few clusters of cells in the altered MH domain, which could explain the observed phenotypes (Sunmonu et al., 2009). Therefore, the correct spatiotemporal juxtaposition of *Otx2*- and *Gbx2*-expressing territories might be essential for the maintenance of *Fgf8* expression and the anterior-to-posterior patterning of the developing MH domain (Sunmonu et al., 2009).

In this line of evidence, ectopic expression of* Gbx2* in the caudal midbrain under the control of the *Wnt1* enhancer caused the creation of a new shared *Otx2*/*Gbx2* interface positioned more rostral than in wild conditions. *Fgf8* and *Wnt1* expressions also shifted and adapted to the newly created *Otx2*/*Gbx2* border (Millet et al., 1999). As a consequence, the midbrain was clearly reduced in these *Wnt1-Gbx2* transgenic mice at early developmental stages. Remarkably, these genetic changes were transient and the *Gbx2* upregulation disappeared at later stages, together with the reestablishment of the *Otx2*/*Gbx2* boundary in its normal position (Millet et al., 1999; see also Li et al., 2005). The changes observed in later stages could be one of the possible reasons by which the adult brains of these mutant mice do not show morphological alterations in the MH domain. In summary, all these words clearly show that both *Otx2* and *Gbx2* genes cooperate in the establishment of the isthmic organizer position, triggering the inductive events mediated by *Fgf8* and *Wnt1* signaling pathways (Broccoli et al., 1999; Millet et al., 1999; Li et al., 2005), confirming and extending previously reported results. Gain/loss of function experiments in *Xenopus* (Tour et al., 2002a, b) and zebrafish (Rhinn et al., 2003, 2009) have added substantial information to the demonstration that most probably *Otx2* is sufficient to activate the MH genetic cascade, while *Gbx2* restricts and defines the sharp *Otx2-*expressing domain, positioning the MH boundary in a dosage-dependent manner.

### *Lmx1b* Genes in the Midbrain-Hindbrain Specification

The *Lmx1b* gene encodes an LIM homeodomain protein with a dynamic expression pattern in the developing central nervous system, including the MH domain (Adams et al., 2000; Liu and Joyner, 2001; Matsunaga et al., 2002; O’Hara et al., 2005; Guo et al., 2007; Kim et al., 2016; Pose-Méndez et al., 2016). In chicks, *Lmx1b* starts to be expressed immediately after *Fgf8* initiation (Adams et al., 2000). At stage HH15–20, *Lmxb1* expression is observed in a band located just rostral to the MH boundary, as defined by the caudal *Otx2* expression, and in the dorsal and ventral midlines of the developing midbrain. The caudal borders of the *Wnt1*- and *Lmx1b*-expressing areas are coincident and abut the *Fgf8*-positive area (Adams et al., 2000; Matsunaga et al., 2002). Using *Fgf8*-soaked beads and retroviral-mediated *Lmx1b* expression (*Lmx1b/RCAS*), it has been reported that *Lmx1b* seems to act as an effector of *Fgf8* in the regulation of *Wnt1* expression (Adams et al., 2000). Thus, *Lmxb1* could be both necessary and sufficient to maintain *Wnt1* expression in the MH domain, regulating the midbrain morphogenesis (Adams et al., 2000). In another line of evidence, *Lmx1b* misexpression by *in ovo* electroporation induced *Wnt1* and *Otx2* expressions, while *Fgf8* expression was repressed (Matsunaga et al., 2002). Ectopic *Fgf8* expression was induced around *Lmx1b*-misexpressing cells. Besides, *Otx2* was able to induce *Lmx1b* expression, whereas *Gbx2* repressed it (Matsunaga et al., 2002). In zebrafish, two *Lmx1b* orthologs, *lmx1b*.*1* and *lmx1b.2*, have been described, with expression patterns similar to those described in chick embryos (O’Hara et al., 2005). Single and double knockdown of *lmx1b*.*1* and *lmx1b.2* have shown that both genes have a redundant function in the specification of the MH domain in fish, these genes being necessary and sufficient for the maintenance of *wnt1* and *fgf8* expressions. Interestingly, Pax2.1 is required for the correct preservation of *lmx1b*.*1* and *lmx1b.2* expression patterns in the developing MH domain in a manner independent of Wnt1 and Fgf8, whereas both *lmx1b* genes are necessary for *pax8* maintenance (O’Hara et al., 2005). All these results clearly suggest that *Lmx1b* may be involved in the positioning of the *Otx2-Wnt1*/*Gbx2-Fgf8* MH boundary in the *Pax2*-expressing competent domain.

**Figure 10:**
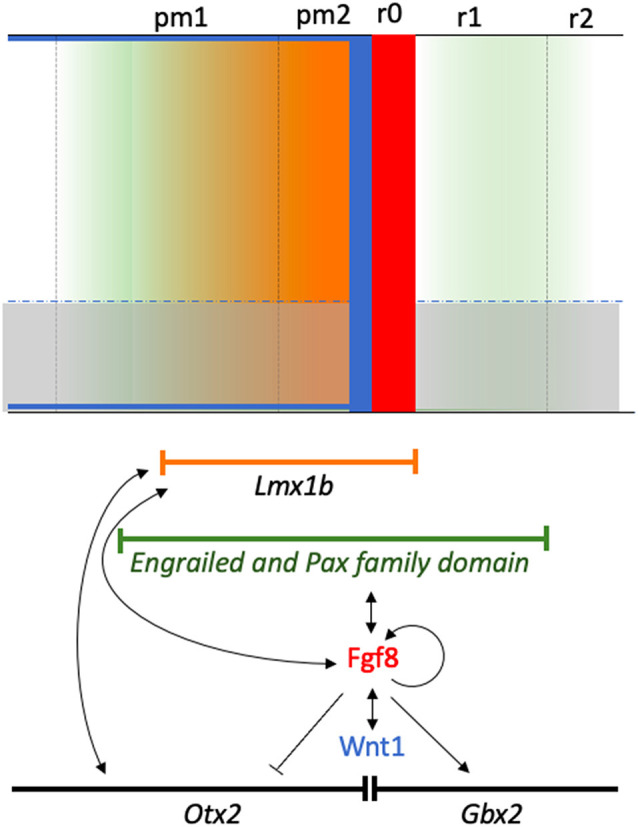
A schematic diagram showing the isthmic neural territory influenced by the morphogenetic activity. The molecular pathways regulating organizer specification are also shown, together with specific local activity associated with the gradient of the signaling molecules (color gradients-color codes). Genetic patterns are represented by their respective symbols inside a lineal sector. Gene interactions are represented by arrows showing the direction of the interaction and inductive effects either positively (arrow) or negatively (no arrow).

In mice, the *Lmxb1* expression patterns displayed some differences with respect to chicks and fish. In mammals, *Lmxb1* expression encompassed the MH boundary, its expressing domain extending to most of the *Fgf8*-expressing area at the 4-somite stage (Guo et al., 2007). The study of *Lmx1b*^−/−^ mutant embryos showed that *Fgf8* expression in the MH domain was fully absent. *Wnt1*, *En1*, and *Pax2* expressions were downregulated prior to the 4-somite stage, while *Gbx2* expression was downregulated at this developmental stage (Guo et al., 2007). Despite the co-expression of *Lmx1b* and *Otx2* genes in the MH domain, *Otx2* expression was not disrupted. This finding suggests that *Lmx1b* is not involved in the positioning of the MH boundary. As a consequence of this changed genetic network, the tectum and cerebellum of *Lmx1b*^−/−^ embryos showed strong alterations, with an appreciable reduction in size. The inferior colliculus was completely lost and the reduced cerebellum contacted with the smaller superior colliculus (Guo et al., 2007). In addition, FGF8 can also induce *Lmx1b* expression in the midbrain explant (Liu and Joyner, 2001). Therefore, Lmx1b could be considered a key factor in the specification of the MH domain in mammals, with a relevant position in the hierarchical genetic cascade governing inductive isthmic activities (Figure 10).

### The Zebrafish *Her5* Gene and Its Vertebrate Orthologous in Patterning and Neurogenesis

The *her5* gene encodes a basic helix-loop-helix (bHLH) protein, homolog of the *Drosophila* hairy-E(spl) family. In zebrafish, *her5* expression is observed in the entire presumptive MH domain at gastrulation (Müller et al., 1996; Tallafuß and Bally-Cuif, 2003). After brain morphogenesis, *her5* transcriptsare detected in the caudal portion of the developing midbrain, its caudal limit being coincident with the MH boundary (Müller et al., 1996). Studies using transgenic embryos carrying the recombined *her5PAC:egfp* construct have shown that the spatiotemporal dynamic expression of zebrafish *her5* governs neurogenesis progression in a converging manner towards the MH constriction (Tallafuß and Bally-Cuif, 2003). During the growth and regionalization of the developing MH domain in zebrafish embryos, the bHLH Hairy/E(spl)-like factor Her5 accurately defines the “intervening zone” (IZ) as a neuron-free transverse stripe of delayed differentiation (Geling et al., 2003, 2004; Tallafuß and Bally-Cuif, 2003), translating early positional information into accurate neurogenesis and cell proliferation in the developing MH domain in collaboration with other E(spl) factors in a dose-dependent manner (Sieger et al., 2004; Ninkovic et al., 2005, 2008; Chapouton et al., 2006; Webb et al., 2011; Galant et al., 2016).

It is worth noting that *Her5* does not affect other aspects of the MH patterning such as the expression patterns of MH markers (*pax2.1*, *eng2*, *eng3*, *wnt1*, and *fgf8*), *her5* being necessary for the maintenance of the integrity of the MH domain but not for the establishment and early maintenance of the MH domain (Geling et al., 2003). In zebrafish *noi* mutant, affecting the *pax2.1* gene, *her5* expression occurs normally (Lun and Brand, 1998). In *acerebellar* mutant embryos, a zebrafish *Fgf8* mutation, *her5* decreases by the 5-somite stage, being absent at the 18-somite stage, suggesting that, similar to *wnt1* and *pax2.1*, *Her5* requires *Fgf8* activity during the maintenance phase of MH development (Reifers et al., 1998). When FGF action decreases along the anterior-to-posterior axis of the developing midbrain as a consequence of the growth of the MH region and an increased distance from the isthmic source of FGF, a posterior retraction of *her5* expression towards the isthmus is detected, together with a loss of Her5-mediated repression of neuronal differentiation (Dyer et al., 2014).

Concerning the WNT signaling pathway, the zebrafish *Dfw5* mutant embryos display deletions in *wnt1* and *wnt10b* genes (Lekven et al., 2003). In *Dfw5* embryos, zebrafish *her5* expression started to be reduced at the 12-somite stage, its expression being undetectable in the ventral portion of the MH domain by 24 hpf (Lekven et al., 2003). Interestingly, *pax2.1* and *en2* expressions were also lost where *her5* was absent in *Dfw5* embryos, whereas *fgf8* and *en3* expressions, as well as those of *wnt8b* and *wnt3a* genes, were not disturbed. Thus, either *wnt1* or *wnt10b* seems to be necessary and sufficient for *pax2*, *en3*, and *her5* maintenance, but not for *fgf8* and *en3* conservation (Lekven et al., 2003; Green et al., 2020). However, *pax2.1* may also play a relevant role in the *her5* regulation (Lun and Brand, 1998).

It is interesting to remark that, in the MH domain, the *her5*-expressing domain forms a sharp border with the *gbx2*-expressing domain, both areas being closely complementary (Nakayama et al., 2017). In *gbx1/gbx2* double homozygotes, *her5* expression expands caudally, which suggests that *her5* could be repressed by *gbx1* and *gbx2* in the rostral-most hindbrain. Also, *her5* expression was downregulated when *gbx2* was overexpressed using mRNA microinjections. Thus, *her5* could participate in positioning of the *otx2*/*gbx2* MH boundary (**N**akayama et al., 2017). Because *her5* negatively regulates neural differentiation in the caudal midbrain, *gbx2* could also promote neurogenesis in the anterior hindbrain by excluding *her5* expression (Nakayama et al., 2017).

Studies of mouse *Hes* genes, orthologous of zebrafish *her* genes, have also reported an evident role of these genes in early midbrain and hindbrain patterning (Lobe, 1997). In E12.5 mice, *Hes3* expression is detected in the caudal midbrain, its expressing domain overlapping the *Wn1*-positive area and abutting the *Fgf8*-positive domain (Hirata et al., 2001). In *Hes1-Hes3* double-mutant embryos, which showed a tendency of growth retardation, the midbrain and the rostral-most portion of the hindbrain are absent. The locus ceruleus (r1) and cranial nerve III and IV were also missing in the double mutant, whereas more caudal cranial nerves (V, VII, IX, and X) were present (Hirata et al., 2001). In these mutant mice, the isthmic organizer cells prematurely differentiate into neurons, together with the progressive loss of expression of MH markers, such as *Fgf8*, *Wnt1*, and *Pax2/5* (Hirata et al., 2001). Besides, loss of FGF activity in the ventral midbrain caused a loss of *Hes1* (Lahti et al., 2011). This finding clearly confirms that *Hes1* and *Hes3* could cooperate to avoid too early differentiation and preserve the isthmic regulatory activities over time (Hirata et al., 2001), probably participating, directly or indirectly, with additional genes in the intricate network of the isthmic organizer. Since *Hes1* expression is activated in Wnt1/PC12 cells, which display a block of differentiation and an increase in proliferation, *Hes1* could be a possible target of the long-term Wnt1 signaling pathway (Issack and Ziff, 1998).

In chick embryos, *Hes5* transcripts were firstly detected in the rostral midbrain at stage 14, where *En2* expression is not strong (Kimura et al., 2004). When development proceeds, *Hes5* expression expanded to the caudal mesencephalon at stage HH20, the isthmic region being clearly *Hes5* negative. As expected, misexpression of *En2* downregulated *Hes5* expression in the mesencephalon. In addition, *Hes5* expression was induced in the isthmus when the siRNA expression vector was used to silence *En2*. Thus, the chick *Hes5* gene could be regulated by En proteins to determine the anterior-to-posterior polarity of the midbrain (Kimura et al., 2004).

The *XenopusHES*-related *1* (*XHR1*) gene encodes a protein homologous to members of the HES family, very close to zebrafish *Her5* (Shinga et al., 2001). *XHR1* expression was detected in the presumptive MH domain in *Xenopus* early gastrula stage (Shinga et al., 2001; Takada et al., 2005; Takahashi et al., 2005), much earlier than *XPax-2, En-2*, *Xotx2*, and *Xgbx2* (Shinga et al., 2001). At tailbud stage, *XFGF-8* expression was included in the *XHR1-*expressing domain. Interestingly, ectopic expression of *XHR1* in the MH domain enhanced *En-2* expression, whereas overexpression of *XHR1* dominant-negative forms caused an evident reduction of *XPax-2* and *En-2* expression (Shinga et al., 2001).

### POU-Domain Transcription Factor Pou2 in the MH Specification

The *Pou2* gene encodes a POU-domain transcription factor. *spiel ohne grenzen* (*spg*) zebrafish mutants, which carry loss-of-function mutations in the *pou2* gene and display alterations in hindbrain segmentation (Hauptmann et al., 2002). In zebrafish *spg/pou2* mutants, the MH boundary is not correctly established (Schier et al., 1996), with an absence of isthmus and cerebellum, the midbrain being reduced in size (Belting et al., 2001). In these *spg* embryos, the trochlear nerve is absent (Reim and Brand, 2002). Regarding the MH genetic cascade, the complementary expression of *otx2* and *gbx1* is normal in *spg* mutant embryos, suggesting that the *pou2* gene is not crucial in positioning the MH boundary. However, it was reported that *gbx2* could be dependent on *spg* function (Rhinn et al., 2003). The initial expression of *pax2.1*, *wnt1*, *her5*, and *eng1/2/3* are severely reduced in *spg* mutants. In an interesting way, *Fgf8* and *Wnt1* signaling activities are activated normally but become dependent on*spg* at the end of gastrulation (Belting et al., 2001; Reim and Brand, 2002). A more severe mutant (*spg^m793^*) displays a complete absence of *fgf8* expression in the isthmus at 24 hpf (Belting et al., 2001). These results suggest that*spg/pou2* could intervene in generating regional competence to respond to Fgf8 activity (Belting et al., 2001; Reim and Brand, 2002). However, *pou2* gene is expressed correctly in *noi* (*pax2.1*) and *ace* (*fgf8*) zebrafish mutants, suggesting that *pou2* does not depend on *fgf8* and *pax2.1* expression (Belting et al., 2001). Nevertheless, the *pou2* could be necessary for the establishment of the *pax2.1*-expressing area in the developing MH domain in a permissive manner (Belting et al., 2001; Burgess et al., 2002). Interestingly, the *pou5f3/pou2* gene, closely related to *Oct4/pou5f1*, is repressed by *gbx2* around the MH domain (Nakayama et al., 2017), being involved in neurogenesis (Inomata et al., 2020).

In mice, the *Oct3/4* (*Pou5f1*) gene, ortholog of the zebrafish *pou2* gene (Burgess et al., 2002), encodes a protein which is able to bind to a functional POU recognition sequence present in the *Pax2* enhancer, suggesting a direct control of *Pax2* expression by one or more members of the POU protein family (Pfeffer et al., 2002). Overexpression of *Oct4* in the neuroectoderm causes a strong upregulation of *Pax2*, suggesting that *Oct4* can regulate *Pax2* during MH development (Ramos-Mejía et al., 2005). A higher dosage of *Oct4* also results in a transient alteration of MH patterning. Thus, *En2* was downregulated at E8,5, its levels of expression being recovered at E9,5. This *En2* alteration could be due to Pax proteins, partly disagreeing with the fact that Pax proteins are involved in the positive regulation of *En2* expression in the developing MH domain (Song et al., 1996). Other regulatory factors should be considered to explain this finding (Parvin et al., 2008). None of the key regulatory factors of the isthmic organizer (*Otx2*, *Gbx2*, *Fgf8*, and *Wnt1*) displayed an altered expression pattern in the dorsal portion of the MH domain. As a consequence, the gross MH morphology was not altered (Ramos-Mejía et al., 2005).

### *bTHS1* and G*rainy Head-Like* Genes as Additional Regulatory Factors

During zebrafish gastrulation, the *bts1* gene, a *btd/Sp1*-related gene, is expressed in the presumptive MH domain before *her5*, *pax2.1*, *wnt1*, and *eng2*, overlapping the *her5*-expressing area and slightly crossing the *otx2* border (Tallafuß et al., 2001). Gain- and loss-of-function experiments have clearly reported that Bts1 is necessary and sufficient for the inductions of *pax2.1*, not being involved in regulating the expression of the other molecular elements of the isthmic genetic cascade, including *fgf8*. Although *bts1* expression was never completely eliminated in the lack of Fgf activity, it probably requires in part the Fgf8 action. Thus, Bts1 seems to be an early upstream component of the inductive pathway leading to the specific induction of the *pax2.1* gene from the mid-gastrulation stages (Tallafuß et al., 2001).

The grainy head-like (Grhl) family of transcription factors are key regulatory elements in several developmental events (Rifat et al., 2010). The zebrafish *grhl2b* and *grhl3* genes are directly involved in the specification and morphogenesis of the MH domain (Dworkin et al., 2012; Miles et al., 2017), probably by means of dissociable events through diverse transcriptional targets (Dworkin et al., 2012). Loss of *grhl2b* expression causes no alteration of *otx2* and *gbx1* expression, suggesting that *grhl2b* is not involved in positioning the MH boundary. However, the expression of MH markers such as *eng2a*, *wnt1*, *pax2a*, and *her5* was lost by 26 hpf, but not before. These results strongly suggest that *grhl2b* participates in the maintenance of the MH domain at both structural and molecular levels. Interestingly, the normal expression patterns of these genes were recovered by re-expression of *eng2a* in *grhl2b* morphants (Dworkin et al., 2012), *eng2a* being a target of the Grh family (Wilanowski et al., 2002). In this sense, *grhl2b* and *fgf8* seem to cooperate to govern the specification and morphogenesis of the MH domain in zebrafish, *grhl2b* probably acting downstream of *fgf8*, through *eng2a* (Dworkin et al., 2012).

### Lineage Restriction at the MH Domain

Descriptive and experimental studies suggested the existence of lineage segregation at the mid- hindbrain junction in birds. Using *Otx2* riboprobe and tissue grafting experiments concerning the formation of the sharp MH boundary, Millet and colleagues showed that the MH constriction prevents the intermingling of midbrain and isthmic cells (Millet et al., 1996). However, more recent experiments in chicks conducted by cell labeling experiments *in ovo* at stages HH11 and HH13 showed that the midbrain and anterior hindbrain could not be separated by cell lineage restriction, with stained cells moving between both tagmas (Jungbluth et al., 2001). A restriction of DiI-crystal labeled cells was observed exclusively in the roof plate of the MH domain (Alexandre and Wassef, 2003; Louvi et al., 2003). In clear contrast, the posterior border of the midbrain *Lrrn1* expression was proposed again to present cell restriction properties; disturbing this compartment-restricted border by ectopical *Lrrn1* expression in the isthmus provoked an alteration of MH organizer genes and a mix of cells from two separated compartments (Tossell et al., 2011a). This *Lrrn1* activity would be mediated by Notch signaling pathways through regulation of the posterior border of *LFng* and *Ser1* expressions (Tossell et al., 2011b).

In mice, the nature of the caudal midbrain and isthmus, i.e., the MH (*Otx2*/*Gbx2*) interface, as entities separated by a lineage restriction boundary was demonstrated using a *Wnt1-CreER^T^* approach as long-term fate mapping, the *Wnt1-CreER^T^*-stained cells being registered with *Otx2* expression (Zervas et al., 2004). These findings were confirmed later when a transgenic mouse line *MHB-Cre* was analyzed, in which a small group of cells was stained at E10.5 next to the caudal border of *Otx2* expression (Kala et al., 2008). Other studies using a *Gbx2^CreER^* knock-in line in mice as a genetic inducible fate mapping showed without any doubt that the *Otx2*/*Gbx2* MH boundary separates proliferating progenitors of the considered contiguous neuromeres, the*Gbx2*-labeled progenies not migrating through it at E7.5 (Sunmonu et al., 2011). In the formation of this lineage boundary, *Fgf8* plays an essential role too; its partial deletion disordered the local organization of cell populations, promoting movements across the MH boundary (Sunmonu et al., 2011). In this sense, *Wnt1-Cre/*+; *Fgfr1^flox/Δflox^* embryos showed that the posterior border of the *Otx2*-expressing domain and the anterior border of the *Gbx2*- and *Fgf8*-expressing domain were significantly altered with a mixture of midbrain and hindbrain cells at this interface (Trokovic et al., 2003). In the analyzed *Fgfr1* mutants, *Wnt1*-positive cells also appear also to mix with *Wnt1*-negative cells, a cellular event in which *PB-cadherin* seems to be involved (Kitajima et al., 1999; Trokovic et al., 2003).

Similar evidence of accurate neuroepithelial patterning and cell differentiation in the MH domain were also reported in the zebrafish MH domain. By staining live embryos with vital dyes and matching the positions of hundreds of stained nuclei within the *Otx2*-labeled domain, it was plainly demonstrated that midbrain and hindbrain (isthmic) cells generate from two separate cell populations during late gastrulation stages (Langenberg and Brand, 2005). Using innovative methodological approaches with several transgenic reporter fish involving MH markers and live imaging, among others, also showed that the lineage restriction taking place along the MH junction is controlled by differential cell-cell adhesion properties in which N-cadherin and Eph-ephrin signaling or even Cx43 based Gap junction might clearly be involved (Bosone et al., 2016; Kesavan et al., 2017, 2018, 2020). Considering all these works, the existence of lineage segregation at the mid- hindbrain junction is confirmed.

### Future Perspectives

In the present review, we provide tons of evidence in which different levels of transcription factors together with signaling molecules are orchestrated and are required to properly develop the specific regions within the embryonic midbrain and cerebellum. It has been also demonstrated that such differences in level and created gradients may underlie the anatomical variations between species. Future studies may reveal and relate the proper levels of these signaling molecules to build different brain regions in lower vertebrate brains. Moreover, we are far from understanding the precise spread mechanisms of these signaling molecules and the exact mechanism of cell-cell communication to acquire specific positional information and proper polarity in order to be a particular brain nucleus and how this communication between embryonic cells in a particular region is propagated.

## Author Contributions

MH-S and DE conceived the research. All authors contributed to the article and approved the submitted version.

## Conflict of Interest

The authors declare that the research was conducted in the absence of any commercial or financial relationships that could be construed as a potential conflict of interest.

## Publisher’s Note

All claims expressed in this article are solely those of the authors and do not necessarily represent those of their affiliated organizations, or those of the publisher, the editors and the reviewers. Any product that may be evaluated in this article, or claim that may be made by its manufacturer, is not guaranteed or endorsed by the publisher.
